# Detoxification therapy of traditional Chinese medicine for genital tract high-risk human papillomavirus infection: A systematic review and meta-analysis

**DOI:** 10.1371/journal.pone.0213062

**Published:** 2019-03-01

**Authors:** Mei Luo, JiaJie Yu, ShuYi Zhu, Li Huang, Yu Chen, ShaoBin Wei

**Affiliations:** 1 Chengdu University of Traditional Chinese Medicine, Chengdu, Sichuan, P.R. China; 2 Department of Gynecology, Hospital of Chongqing Institute of Traditional Chinese Medicine, Chongqing, P.R. China; 3 Chinese Evidence-Based Medicine Center, West China Hospital, Sichuan University, Chengdu, P.R. China; 4 Hospital of Chengdu University of Traditional Chinese Medicine, Chengdu, Sichuan, P.R. China; Stanford University School of Medicine, UNITED STATES

## Abstract

**Background:**

Persistence of high-risk human papillomavirus (hr-HPV) infections is the most critical risk factor for cervical intraepithelial neoplasia (CIN) and cervical cancer (CC). Treatment of persistent oncogenic HPV-positive women after 12–24 months follow-up is still controversy. Detoxification therapy of Chinese medicine (DTCM) has been conducted recently. However, the conclusions are still unclear. We planned to conduct a systematic review and meta-analysis to explore DTCM in the treatment of persistent hr-HPV infections.

**Methods:**

Nine electronic databases were systematically searched from their inception to 30 September 2018. Randomized controlled trials comparing DTCM with follow-up or placebo were included. Risk of bias was assessed by the Cochrane ‘Risk of Bias’ tool. Review Manager 5.3 was used for statistical analyses. Relative ratios (RR) and 95% confidence intervals were used for dichotomous data, and the mean difference (MD) was used for continuous data. We assessed the quality of trials by the GRADE.

**Results:**

Seventeen RCTs from 2011 to 2018 with 1906 participants were included. The evidence showed that DTCM had a pooled efficacy difference in favor of increasing the HPV clearance rate compared to placebo groups (RR = 2.62, 95% CI 1.28 to 5.33, very low quality) and follow-up groups (RR = 1.88, 95% CI 1.60 to 2.22, low quality). The median HPV persistence tended to decline from 50% within six months to 41.5% at 12 months, and 31.5% at 24 months. A significantly increased regression rate of CIN was found in the DTCM compared with placebo groups (RR = 3.61, 95% CI 1.21 to 10.83, very low quality) and follow-up groups (RR = 1.79, 95% CI 1.31 to 2.45, very low quality). Additionally, we found DTCM have an impact on TNF-α (MD = 2.99, 95% CI 1.90 to 4.07; very low quality), IFN-α (MD = 3.47, 95% CI 2.42 to 4.52; very low quality), CD^4+^/CD^8+^ cells (MD = 0.21, 95% CI 0.05 to 0.37; very low quality) compared with follow up groups in some trials with small sample sizes. The major adverse events were genital mucosal irritation symptoms (10%, 5/50).

**Conclusions:**

DTCM have favorable outcomes on improving the HPV clearance rate, increasing the regression rate of CIN, and impacting the proportion of some immune cells and cytokine levels. However, most of the evidence was of low quality. Any future high-quality trials and a more extended follow-up period of 24 months or more should be performed.

## Introduction

High-risk human papillomavirus (hr-HPV) infection is the primary risk factor of cervical cancer (CC) and its precancerous cervical intraepithelial neoplasia (CIN) [1, 2]. It is one of the six human viruses which has been classified by the International Agency for Research on Cancer (IARC) as Group 1 (carcinogenic to humans) [3]. Although hr-HPV infection is prevalent in sexually active women, typically transient and cleared within months to 2 years [4, 5], a few cervical infections still persistent and progress to CC. A prospective study among 3282 women in the Netherlands reported that approximately 34% of young women fail to clear HPV in 2 years [6]. In mainland China, the reported persistent infection rate among women (ages 16–69) was 13.30–22.94% [7–9].

The treatments on cervical HPV infection were limited. The American Society for Colposcopy and Cervical Pathology (ASCCP) guidelines recommend that women with “lesser abnormalities”, which include hr HPV-positive but cytology-negative (or cytology-atypical squamous cells of undetermined significance (ASC-US), or low-grade squamous intraepithelial lesions (LISL), should be managed by hr-HPV genotyping and follow-up in 12 months [10]. However, the risk of malignant progression would be increased by persistent carcinogenic infection according to recent studies [11]. If hr-HPV infection persistent for more than one year, 21% of women progressed to CIN2 or got higher [12]. The intervention of oncogenic HPV-positive people has been the focus of controversy. Some scholars suggested cryotherapy to be one of the treatment options for LSIL, for a cohort study in Taiwan found that cryotherapy for women with LISL could reduce the occurrence rate of CIN3+ by improving HPV infection clearance [13].

There is no corresponding description consistent with genital tract hr-HPV infection disease in the ancient medical records of Traditional Chinese medicine (TCM). According to several symptoms such as vaginal discharge abnormalities and cervical contact bleeding in some patients, the cervical disease was described as leucorrhea disease and colporrhagia during sexual intercourse. According to the TCM theory, the syndrome is thought to be caused by toxin invasion. Chinese medical physicians usually use detoxification therapy alone or plus a series of treatments such as dispelling dampness and replenishing qi therapy to treat the symptoms. Several trials have shown the role of detoxification therapy of Chinese medicine (DTCM) in the treatment of HPV infection. In those trials, DTCM appears to be effective and safe for HPV infection and CIN. However, the optimal treatment strategy was still controversy. Currently, there are no systematic reviews of this subject. Therefore, we conducted a systematic review and meta-analysis, aiming to offer a comprehensive assessment of DTCM as an adjunctive therapy on the treatment of persistent hr-HPV infections.

## Materials and methods

We followed the reporting standards for systematic reviews and meta-analyses of randomized controlled trials according to PRISMA statements. The protocol has been registered with the Prospective Registration of Systematic Reviews (PROSPERO) (Number CRD42018092712).

### Study selection

We included prospective, randomized controlled trials (RCTs) if they include women diagnosed with persistent oncogenic HPV-positive but cytology-negative (or ASC-US, or LISL); compared DTCM (treatment duration up to 3 months) with placebo or follow up. The definition, screening of hr-HPV and cervical cytology was according to ASCCP [10], the American College of Obstetricians and Gynecologists (ACOG) guidelines [12] and Chinese Society for Colposcopy and Cervical Pathology of China Healthy Birth Science Association (CSCCP) guideline [14]. Hr-HPV persistent infection is defined as the infection last one year or more, and the same hr-HPV subtype was positive in retesting at intervals of the 6–12 months [12].

We excluded trials with the following details: cross-over trials and quasi-randomized trials; trials with the undistinguishable type of hr-HPV or lr-HPV; participants diagnosed during pregnancy, or with high-grade CIN 2, 3+; and, TCM combined with interferon, acupuncture or TCM fumigation therapy were also excluded. We also excluded studies for which the original data could not be extracted, even if we had contacted the author.

### Types of outcome measures

#### Primary outcome

The rate of hr-HPV clearance (the definition of clearance is negative results for type-specific HPV for two subsequent study visits every 6–12 months), the regression rate of CIN at different follow-up time points.

#### Secondary outcome

The proportion of immune cells in the genital tract, changes in cytokine levels, adverse events, and the rate of reoccurrence.

### Data source and searches

Cochrane Controlled Trials Register, Cochrane Reviews, Medline, Embase, Web of Science, Chinese Biomedical Databases, China National Knowledge Infrastructure (CNKI), Chinese Scientific Journals Database (VIP), and Wanfang data were searched from their inception to 30 September 2018 [15]. Mesh and keyword search terms included were “East Asian Traditional Drugs”, “Alternative medicine”, “HPV” (human Papillomavirus infections), “ASC-US”, “LSIL”, “zhong yi yao” (traditional Chinese medicine), “zhong yi” (Chinese medicine), “zhong yao” (Chinese herbs), “zhong cheng yao” (Chinese patent medicine). We also searched other resources as following: the WHO International Clinical Trials Registry Platform (http://www.who.int/ictrp/en/), Clinical Trials.gov (https://www.clinicaltrials.gov/), Chinese Clinical Trial Registry (http://www.chictr.org.cn/), and the reference list of the retrieved articles. No limitation about publishing language.

### Data collection

Two review authors (ML and SYZ) screened and extracted data independently. Any disagreement was resolved through discussion. We included information about publication year, the total number of participants, age, type of hr-HPV, screening methods, interventions and control measures (dose, route, length of therapy), outcome measurement, and different follow-up time points.

### Risk of bias

We assessed the risk of bias using the Cochrane ‘Risk of Bias’ tool [16], which included selection bias (random sequence generation, allocation concealment), performance bias (blinding of participants and personnel), detection bias (blinding of outcome assessment), attrition bias (incomplete outcome data), reporting bias (selective reporting), and other bias (other sources of bias). We assigned a grade for each domain as low, high, or unclear for risk of bias. Any disagreement was resolved by consensus.

### Data analysis

We used Review Manager 5.3 for statistical analyses [16]. The relative ratios (RR) was used to calculate the treatment effect with 95% confidence intervals (95%CIs) for dichotomous data (e.g., HPV cleared or non-cleared). The mean difference (MD) was used to calculate the treatment effect for continuous data (e.g., levels of immune cells). If possible, we conducted analysis based on intention to treat (ITT). We applied the random-effects model using the Mantel-Haenszel method to pool the data. We used the t^2^ and Chi^2^ statistics to analyze the heterogeneity between trials (the test level was set to α = 0.05). Funnel plot by visually inspected was used to investigate publication bias when more than ten trials were included.

We planned a pre-specified subgroup variable (length of follow up) to explore the heterogeneity. An interaction test was used to test the subgroup difference. Sensitivity analysis (omitting one individual study might have a significant effect on the heterogeneity at the time with recalculation) was used to investigate whether the conclusions were robust.

### Grading the quality of evidence

The GRADEpro Guideline Development Tool [17] was used to import data from Review Manager 5.3 to create a ‘Summary of Findings’ table [16]. We used bias risk, inconsistency, evidence indirectness, inaccuracy, and publication bias as criteria to assess the quality of evidence for each outcome and assign four quality levels: high, moderate, low, and very low. If the RCT was defective, we downgraded the quality of the evidence by one to two levels.

## Results

**Fig 1:**
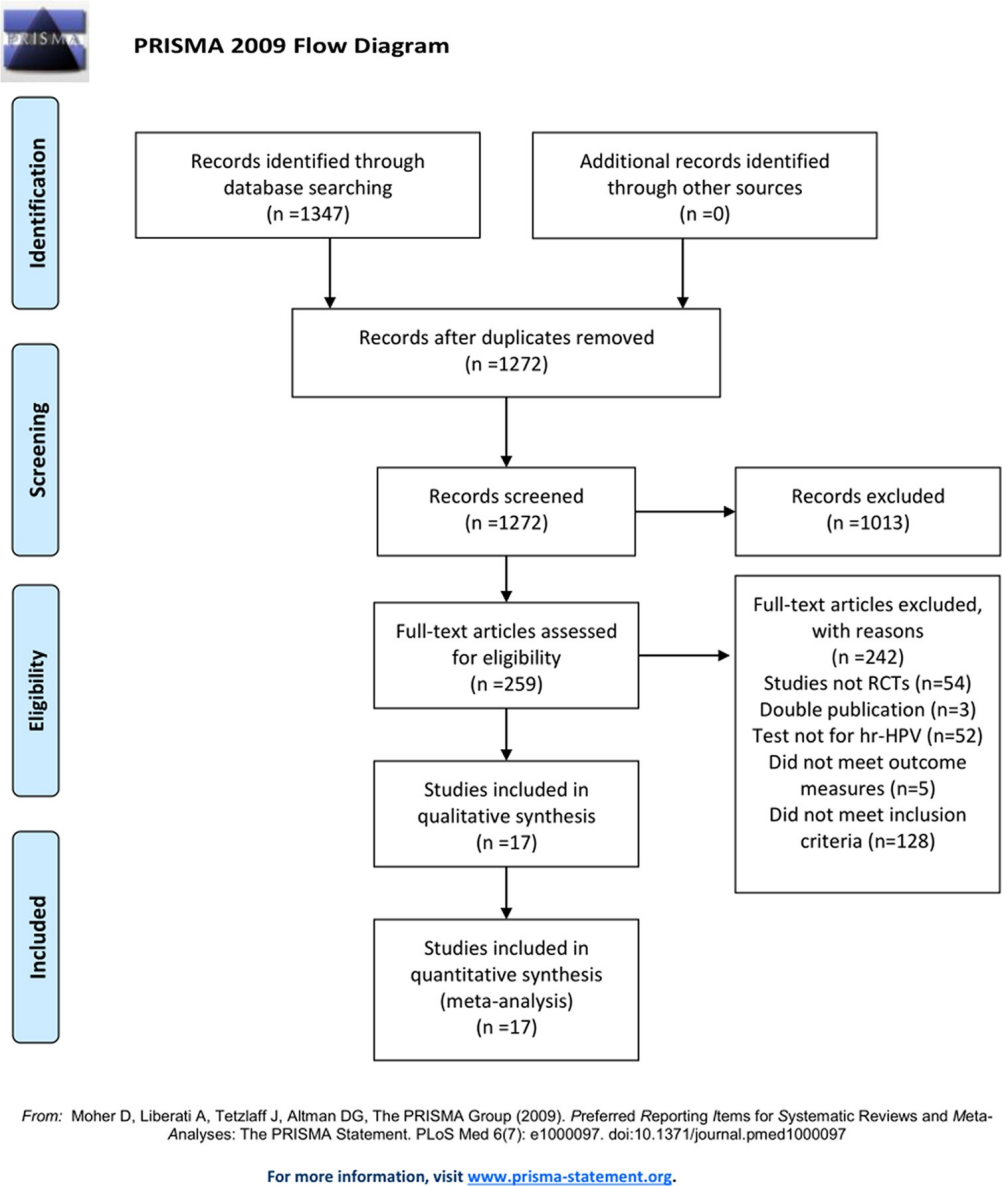
Flow diagram for the process of identifying eligible randomized controlled trials.

The initial search of electronic databases retrieved 1347 trials. After title and abstract reading, 259 trials were potential eligibility; 17 trials published in Chinese from 2011 to 2018 were identified finally [18–34] after reading the full text (Fig 1).

Seventeen RCTs including 1906 women were identified (1036 for the treatment group, and 870 for the control group). Fourteen trials [18, 20, 22–25, and 27–34] compared DTCM with follow-up, and three compared with placebo [19, 21, 26]. Twelve trials (71%) [18, 19, 23, 25–27, and 29–34] used HC-2 (hybrid capture-2), and five (29%) [20–22, 24, and 28] used PCR (Polymerase chain reaction) with or without Gene-chip as the screening methods for hr-HPV. All of 17 trials used TCT (Thin Prep cytology test) as the screening methods for cervical pathology. Eleven trials [19–24, 27, 30–32, and 34] combined with colposcopy. Two trials [28, 34] reported the specific hr-HPV types and the number of infected cases. One trial [24] reported mixed infections of hr-HPV and lr-HPV. Ten trials (59%) [18–22, 24, and 31–34] used a DTCM compound decoction /pill alone or combined with a vaginal preparation of the Chinese herbal compound. The rest of those used the Baofukang Suppository (which mainly included Borneol and Curcuma oil). The follow-up time points ranged from 3 to 24 months (Table 1).

**Table 1:** Characteristics of included trials [ordered by study ID].

Study ID (First Author, Year)	Total population	Age (mean ± SD) (years)	Screening Method for HPV	Screening Method for CIN	Type of hr-HPV	Interventions		Rate of hr-HPV clearance		Follow-up time points (months)	Outcomes ^a^
						Treatment group (drug/dosage/frequency)	Control group	Treatment group	Control group		
Lou JY 2011[18]	60	T:32.23±7.24 C:32.56±7.18	HC-2	TCT	N/A	Qingdu Vaginal Suppository,570mg,qod	Follow-up	36.70%	16.70%	3	1;4
Xiao J 2011[19]	47	T:33.08±7.73 C:34.18±7.86	HC-2	TCT, Colposcope	N/A	Youdujing external lotion,100ml,Twice a week; Youdujing cream, Twice a week	Placebo	58.80%	40.00%	3,6	1;2
Yan X 2012[20]	65	T:37.97±9.10 C:34.94±9.14	PCR	TCT, Colposcope	N/A	Zhidai tablet,po,12 pills/d	Follow-up	53.10%	36.40%	3,8	1;3
Xiao J 2012[21]	70	T:34.20±7.90 C:33.0±7.0	PCR	TCT, Colposcope	N/A	Youdujing external lotion,100ml,Twice a week; Youdujing cream, Twice a week	Placebo	62.10%	16.70%	3	1;2
Zhang J 2012[22]	75	T:35.98±8.97 C:34.33±9.58	PCR	TCT, Colposcope	N/A	Beixiezhidai tablet,po,12 pills/d	Follow-up	43.90%	20.60%	3	1;3
Shen JJ 2013[23]	226	T:31.78±6.54 C:33.59±7.41	HC-2	TCT, Colposcope	N/A	Baofukang Suppository,3.48g,qd	Follow-up	80.22%	60.00%	6,12,24	1;2
Xu YX 2013[24]	100	20–53	PCR+ Gene-chip method	TCT, Colposcope	Hr-HPV in combination with low-risk HPV	Erhuang Vaginal Powder, N/A, qod	Follow-up	58.00%	16.00%	3,6,9	1;2;4
Zhang H 2013[25]	110	T:30.00±7.00 C:31.40±4.98	HC-2	TCT	N/A	BaofukangSuppository,3.48g,qd; Gubenjiedu Decoction, po, bid	Follow-up	68.60%	32.50%	6	1;4
HuangWF 2014[26]	80	25–56	HC-2	TCT	N/A	Gongjingkang Vaginal gel,1.0g,qd	Placebo	28.00%	0%	3,12	1;4;5
Shen JF 2014[27]	106	T:46.00±1.00 C:45.5±0.50	HC-2	TCT, Colposcope	N/A	Baofukang Suppository,1.74g,qd	Follow-up	37.70%	20.80%	3	1
Zhao J 2015 [28]	243	T:37.03±9.33 C:35.82±9.31	PCR, Gene-chip method	TCT	HPV16,18, Other types	BaofukangSuppository,3.48g,qd	Follow-up	HPV16:70.73%; HPV18:70.00%; Other types:70.06%	HPV16:50.00% HPV18:55.56%; Other types:44.0%	4,8	1;4
Wang XS 2015[29]	106	T:46.00±1.00 C:45.5±0.50	HC-2	TCT	N/A	BaofukangSuppository,1.74g,qd	Follow-up	58.49%	18.87%	3	1
Chen YL 2016[30]	200	25–55	HC-2	TCT, Colposcope	N/A	BaofukangSuppository,1.74g,qd	Follow-up	43.00%	19.00%	3	1
Xu CQ 2017[31]	251	T:34.9±6.1 C:35.1±5.9	HC-2	TCT, Colposcope	N/A	Chinese herbal compound Decoction, po, qd; BaofukangSuppository,3.48g,qd	Follow-up	71.50%	56.20%	12,24	1;2
Liu R 2018[32]	40	18–54	HC-2	TCT, Colposcope	N/A	Erhuang Vaginal Suppository,N/A,qod	Follow-up	85.00%	0%	3,9	1;2
Wen LJ 2018[33]	70	T:35.4±6.51 C:34.0±6.23	HC-2	TCT	N/A	Chinese compound herb Vaginal Powder, 3g, qod	Follow-up	54.29%	20.00%	4,12	1;4
Xia N 2018[34]	57	T:39.55±5.17 C:39.21±5.45	HC-2	TCT, Colposcope	HPV16,18, Other types	Ermiao Decoction, po, bid	Follow-up	65.52%	21.43%	6	1;2;3;4

**Abbreviations: T**, Treatment group; **C**, control group; **HC-2**, hybrid capture-2; **PCR**, polymerase chain reaction; **TCT**, thin prep cytology test; **N/A**, no detailed information.

**a:** Include the following: mortality(1), Rate of hr-HPV clearance;(2), Regression rate of CIN; (3), Proportion of immune cells; (4), Adverse events; (5), Rate of reoccurrence.

The risk of bias was moderate or high in most of the evaluation entries. Two trials described the stochastic methods in detail as random numbers [26, 29]. One trial [26] used a central random allocation scheme as mentioned allocation. Three trials [19, 21, 26] were designed with blinding of participants and personnel. Four [19, 21, 23, and 24] reported data missing; none of those trials indicate their utilization with intent-to-treat (ITT). Protocols of all included trials were not identified. All risk of bias was summarized in S2 Table.

### DTCM treatment vs. follow-up

#### The rate of HPV clearance

**Fig 2:**
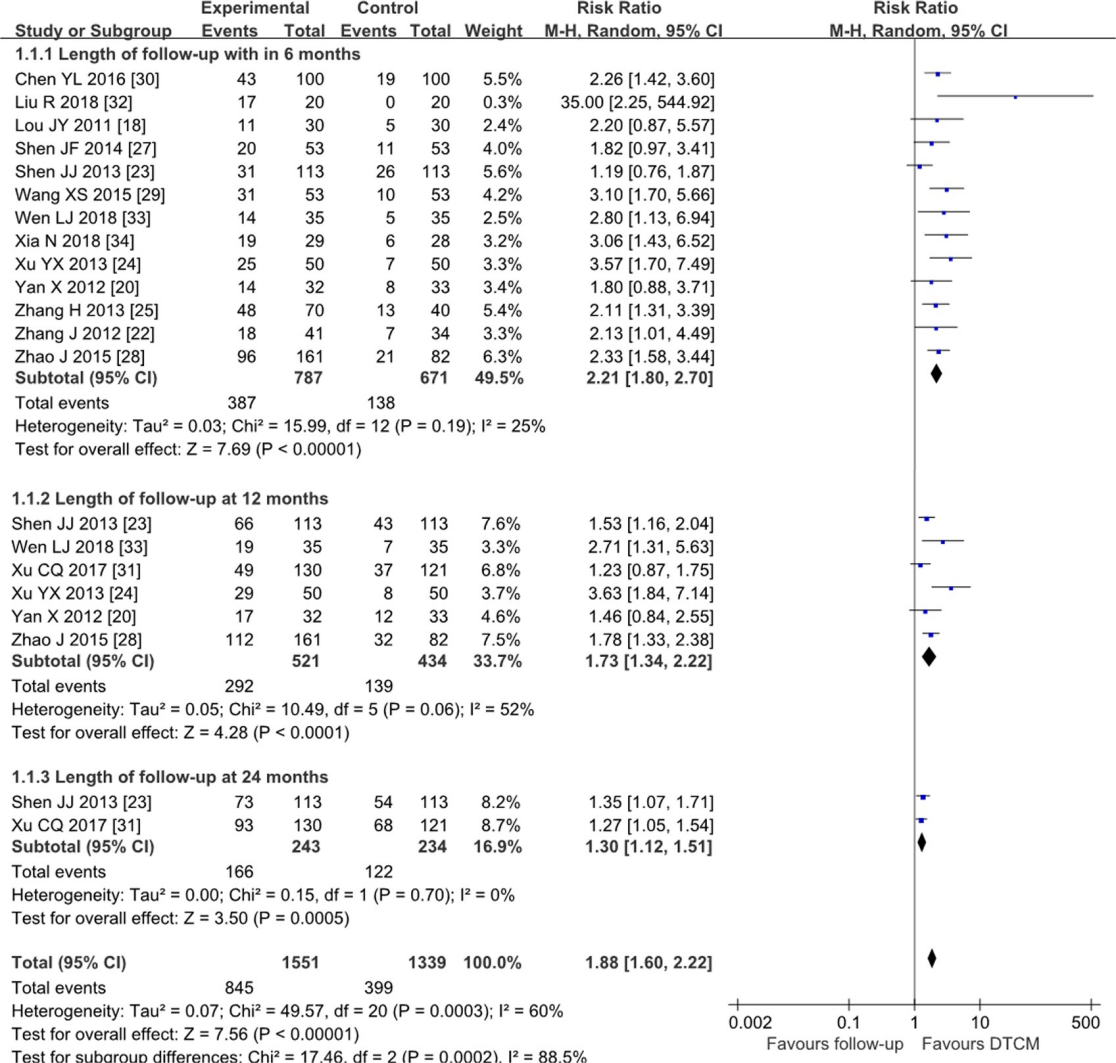
Analysis 1.1 Forest plot of comparison: DTCM treatment alone compared with follow-up. Outcome 1.1: Rate of hr-HPV clearance.

Fourteen trials [18, 20, 22–25, and 27–34] (n = 1709) reported the HPV clearance rate. The results indicated that DTCM groups significantly improved the rate of HPV clearance comparing to follow-up groups (P = 0.0003), (RR = 1.88, 95% CI 1.60 to 2.22, I^2^ = 60%, low quality) (Fig 2, Analysis 1.1). The pooled efficacy of DTCM for persistent hr-HPV women tended to decrease with increasing follow-up time. The most apparent increasing difference was within six months after the end of treatment but gradually approached the control groups at 24 months. The subgroup analysis suggested a significant difference (interaction P = 0.0002). The funnel plot showed some asymmetry.

#### The regression rate of CIN

**Fig 3:**
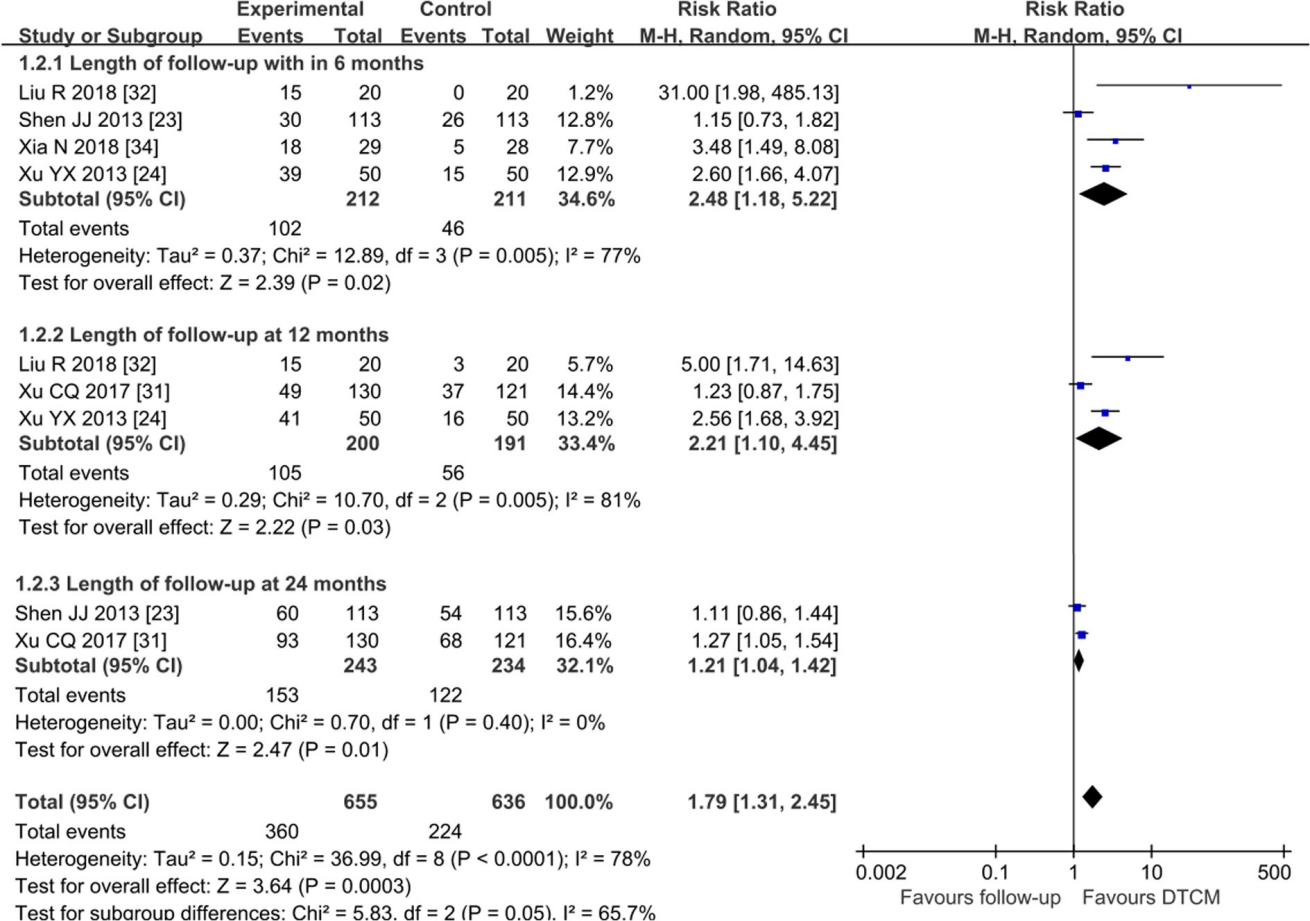
Analysis 1.2 Forest plot of comparison: DTCM treatment alone compared with follow-up. Outcome 1.2: Regression rate of CIN.

Five trials [23, 24, 31, 32, and 34] (n = 674) showed the regression rate of CIN in the results. The results indicated that DTCM improved the regression rate of CIN comparing to follow-up groups (P<0.0001), (RR = 1.79, 95% CI 1.31 to 2.45, I^2^ = 78%, very low quality) (Fig 3, Analysis 1.2). Three subgroup analyses were carried out for different lengths of follow-up by cervical cytology retesting. The subgroup analysis suggested no difference (interaction P = 0.05).

#### Impact on the level of TNF-α in the genital tract

Two trials [20, 22] (n = 140) reported the impact on the level of TNF-α after treatment. The results indicated that DTCM groups have significantly increased the levels of TNF-α (P<0.00001), (MD = 2.99, 95% CI 1.90 to 4.07, I^2^ = 0%, very low quality). Two subgroup analyses were carried out for different lengths of follow-up. The subgroup analysis suggested no difference (interaction P = 0.55). (S1 Fig)

#### Impact on the level of IFN-α in the genital tract

Two trials [20, 22] reported the impact on the level of IFN-α compared with follow-up. The analyses results indicated that DTCM has significantly increased the levels of IFN-α (P<0.00001), (MD = 3.47, 95% CI 2.42 to 4.52, I^2^ = 0%, very low quality). Two subgroup analyses were carried out. We found that the result at eight months indicated a lower level than at three months, but still higher than a follow-up group (P<0.0001), (MD = 3.81, 95% CI 2.08 to 5.54). The subgroup analysis by different lengths of follow-up suggested no difference (interaction P = 0.63). (S2 Fig)

#### Impact on the proportion of immune cells

Only one trial [34] (n = 65) reported the impact on the proportion of CD4+/CD8+ cells compared with follow-up at six months. The results indicated that DTCM was small but significantly increased the ratio of CD4+/CD8+ cells at three months (P<0.01), (MD = 0.21, 95% CI 0.05 to 0.37, very low quality) (S3 Fig).

#### Adverse events

Seven trials [18, 24–26, 33, and 34] observed the adverse events in treatments. Only one trial (DTCM vs. follow-up) [24] reported five cases (10%, 5/50) of genital mucosal irritation symptoms related to DTCM.

### DTCM treatment vs. placebo

#### The rate of HPV clearance

**Fig 4:**
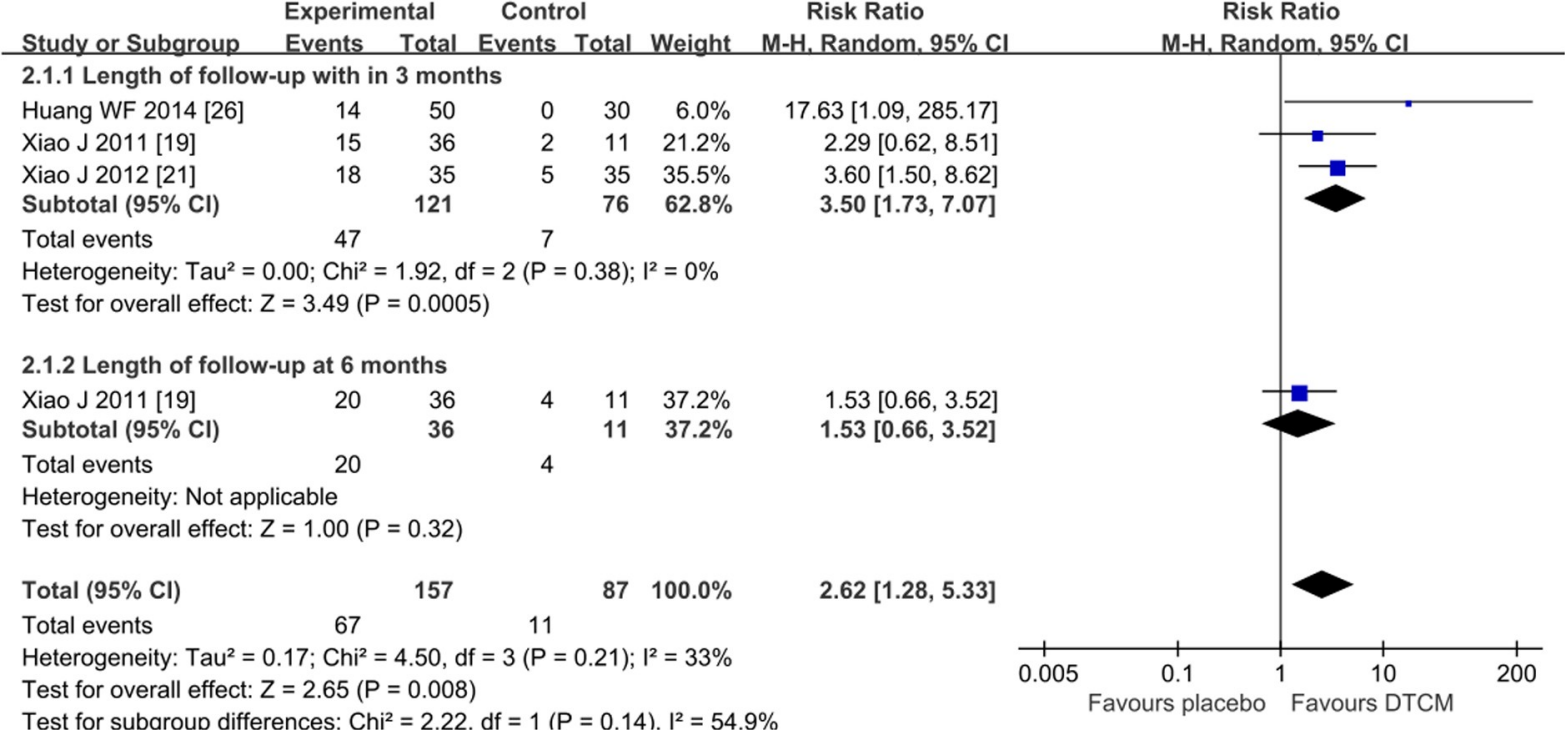
Analysis 2.1 Forest plot of comparison: DTCM treatment alone compared with placebo. Outcome 2.1: Rate of hr-HPV clearance.

Three trials [19, 21, and 26] (n = 197) reported the HPV clearance rate. The results indicated that DTCM significantly improved the rate of HPV clearance (P = 0.0008), (RR = 2.62, 95% CI 1.28 to 5.33, I^2^ = 33%, very low quality). We found no difference between DTCM and placebo groups at six months (P = 0.32), (RR = 1.53, 95% CI 0.66 to 3.52). The subgroup analysis by different lengths of follow-up suggested no difference (interaction P = 0.14). (Fig 4, Analysis 2.1)

#### The regression rate of CIN

Two trials [19, 21] (n = 150) reported the regression rate of CIN. The results indicated that DTCM significantly improved the regression rate of CIN (P = 0.02), (RR = 3.61, 95% CI 1.21 to 10.83, I^2^ = 35%, very low quality). Two subgroup analyses were carried out by different lengths of follow-up. We found no significant difference between DTCM and placebo at six months (P = 0.17), (RR = 2.04, 95% CI 0.74 to 5.58). The subgroup analysis suggested no difference (interaction P = 0.19). (S4 Fig)

#### The rate of reoccurrence

Only one trial [26] reported the rate of reoccurrence. The DTCM group had lower reoccurrence [14.00%, (7/50)] than a placebo group [43.33%, (13/30)] at the end of 12 months follow-up. However, the types of hr-HPV in this trial were not mentioned. These had made it difficult for the author to judge whether it was recurrence or re-infection. We recommend caution in interpreting these results.

### Sensitivity analyses

We removed the outlier trial Liu R2018 [32] in sensitivity analyses of the HPV clearance rate within six months (DTCM vs. follow-up), there was a negligible impact on the random effects pooled efficacy difference (RR = 1.85, 95% CI 1.58 to 2.16, I^2^ = 56%). Similar results were found in other outcomes analyses where the trial by Liu R2018 was excluded.

### Quality of the evidence

For all comparisons (DTCM versus follow-up, DTCM versus placebo), the quality of evidence ranged from low to very low (the rate of hr-HPV clearance; the regression rate of CIN; and the impact on the level of TNF-α, IFN-α, and CD4+/CD8+cells were analyzed). The main limitations were a severe risk of bias, serious inconsistency, and serious imprecision. We have a summary of all findings in S3 Table.

## Discussion

This study systematically reviewed the existing evidence about investigating the efficacy of DTCM in the treatment of persistent hr-HPV infections. Currently, treatment of persistent oncogenic HPV-positive women after 12–24 months follow-up has been the focus of controversy. In addition to its advantages of more abundant sources and lower prices, Chinese material medicine is a critical component of TCM and has been practiced for thousands of years in China. Many molecules extracted from herbs and natural products have been found in the laboratory to be potential to prevent cancer [35]. Chinese medicine was reported to block the expression of E6, E7 gene in HPV16, regulating immune function in cell and animal study [36]. However, epidemiological literature regarding whether DTCM effectively cleans hr-HPV and prevents CC from happening is limited.

This meta-analysis of 17 RCTs with a total of 1906 participants showed that DTCM appeared to improve the HPV clearance rate and regression rate of CIN. The increased probability of HPV clearance was 1.8-fold relative to “follow-up groups”, and 2.6-fold relative to placebo. The regression rate of CIN was 3.6-fold relative to placebo, and 1.8-fold relative to “follow-up groups”. Median HPV persistence tended to decrease with increasing follow-up time, declining from (50%/787 subjects) within six months after treatment to (41.5%/521 subjects) at 12 months, and (31.5%/243 subjects) at 24 months. However, we found the pooled efficacy of DTCM for persistent hr-HPV clearance tended to decrease with increasing follow-up time. The most apparent increasing difference was within six months after the end of treatment but gradually approached the follow-up (or placebo) control groups at 24 months.

To the best of our knowledge, there is no meta-analysis about investigating the efficacy of DTCM for women with persistent hr-HPV but cytology-negative (or ASC-US, or LISL). To high-grade CIN (CIN 2,3), evidence showed ablative or excisional treatment could reduce the median HPV persistence [37]. They showed the downward trend of median HPV persistence declined from 21% at six months to 10% at 24 months. In this meta-analysis, the efficacy of DTCM was lower than treatment in removing HPV infected cells. But it is worth mentioning that the evidence showed a similar downward trend. In other words, the aim of our study was more concerned with the efficacy of DTCM conservative treatment of low-grade cervical lesions with persistent hr-HPV infection. However, fewer trials in this review had long-term (more than 24 months) follow-up. Additionally, we found DTCM has an effect on some immune cells and cytokine in some trials with small sample sizes. We speculated that DTCM might achieve therapeutic goals by improving immune function over a long-term.

There are several limitations in our meta-analysis. First, most of the including trials had a high or unclear risk of bias, making the quality of evidence was low or very low in all comparisons. Heterogeneity within some of the subgroups remained high. Second, the definitions of persistent hr-HPV infection, HPV clearance, and the HPV test methods varied greatly across trials. Some studies did not mention more details about the convention’s definition. We recognized that there would be heterogeneity in definitions between trials. Furthermore, we were unable to perform subgroup analyses according to the specific type of HPV since this information was not thoroughly reported in the included trials. Which also made it difficult to judge whether it was recurrence or re-infection. Third, the specific drugs included in our meta-analysis were mainly Chinese herbal compound decoctions and suppositories, and the herbal ingredients and routes of administration were various. We were unable to perform subgroup analyses by the specific components of DTCM since few trials using identical herbal. Although according to the characteristics of TCM holistic view and syndrome differentiation, similar treatment therapy can lead to a similar efficacy. We still recognized that there would be heterogeneity in interventions between trials. As the number of RCTs increases, we will conduct subgroup analysis by specific TCM herbal in the future. Fourth, due to review-level limitations, all of the included RCTs were carried out in China, reporting bias may exist. Finally, only two trials with a total of approximately 477 subjects provided data at 24 months. While the sample size is probably adequate for a common infection such as hr-HPV and common endpoints such as clearance, it does mean that few trials were contributing to the review.

## Conclusions

Detoxification therapy of Chinese medicine appears to have favorable effects on improving the rate of HPV clearance, increasing the regression rate of CIN, and impacting the proportion of some immune cells and cytokine levels in the genital tract after treatment. The median HPV persistence tended to decrease with increasing follow-up time. However, due to the poor methodological quality and high heterogeneity of the included trials, our conclusions should be carefully interpreted. Any future high-quality evidence trials should elaborate with a rigorously designed large study sample method and determine both the cost-efficacy of different interventions and the impact of DTCM on the quality of life of patients. A longer follow-up period of 24 months or more should be performed in the future.

## Supporting information

S1 Table PRISMA DTA checklist for the title “Detoxification therapy of traditional Chinese medicine for genital tract high-risk human papillomavirus infection: A systematic review and meta-analysis”. (DOC)

S2 Table Risk of bias of included trials: Review authors’ judgments about each risk of bias item for included trials. (DOCX)

S3 Table Summary of findings. (DOCX)

S1 File Electronic search strategy. (DOCX)

S2 File Minimal underlying data of all included studies. (DOCX)

S1 Fig Forest plot of comparison: The impact on the level of TNF-α by DTCM treatment virus follow-up groups. (TIF)

S2 Fig Forest plot of comparison: The impact on the level of IFN-α by DTCM treatment virus follow-up groups. (TIF)

S3 Fig Forest plot of comparison: The impact on the proportion of CD4+/CD8+ cells by DTCM treatment virus follow-up groups. (TIF)

S4 Fig Forest plot of comparison: Regression rate of CIN by DTCM treatment virus placebo. (TIF)
