# Supplementary material for: Detoxification therapy of traditional Chinese medicine for genital tract high-risk human papillomavirus infection: A systematic review and meta-analysis
Source: PLoS One. 2019 Mar 1;14(3):e0213062. doi: 10.1371/journal.pone.0213062 (PMC6396931; doi:10.1371/journal.pone.0213062)
Supplement: S2 Table — (DOCX) [file pone.0213062.s002.docx]

**S2 Table. Risk of bias of included trials: review authors’ judgments about each risk of bias item for included trails.**

| **Study ID**  **(First Author, Year)** | **Authors’ judgment and supporting** **about each risk of bias** | | | | | | |
| --- | --- | --- | --- | --- | --- | --- | --- |
|  | **Random sequence generation** | **Allocation concealment** | **Blinding of participants and personnel** | **Blinding of outcome assessment** | **Incomplete outcome data** | **Selective reporting** | **Other bias** |
| LouJY2011[18] | U  [Due to not reported more random sequence generated details] | U  [Not stated] | H  [Due to no blinding] | U  [Not stated] | L  [Due to all women randomized were analyzed] | U  [ Due to unable to determine prespecified outcomes] | None |
| Xiao J2011[19] | U  [Due to not reported more details] | U  [Not stated] | L  [Due to use blinding of participants] | U  [Not stated] | H  [Due to 32%(23/70)of data missing] | U  [No study protocol] | None |
| Yan X2012[20] | U  [Due to not reported more details] | U  [Not stated] | U  [Not stated] | U  [Not stated] | L  [Due to completeness of data was adequate] | U  [No study protocol] | None |
| Xiao J2012[21] | U  [Due to not reported more details] | U  [Not stated] | L  [Due to use blinding of participants] | U  [Not stated] | L  [Due to15%(11/70)of data missing,] | U  [No study protocol] | None |
| Zhang J2012[22] | U  [Due to not reported more details] | U  [Not stated] | U  [Not stated] | U  [Not stated] | L  [Due to completeness of data was adequate] | U  [No study protocol] | None |
| Shen JJ2013[23] | U  [Due to not reported more details] | U  [Not stated] | U  [Not stated] | U  [Not stated] | L  [Due to16.8% (38/226) of data missing] | U  [No study protocol] | None |
| Xu YX2013[24] | U  [Due to not reported more details] | U  [Not stated] | U  [Not stated] | U  [Not stated] | L  [Due to12%(12/100)of data missing] | U  [No study protocol] | None |
| Zhang H2013[25] | U  [Due to not reported more details] | U  [Not stated] | U  [Not stated] | U  [Not stated] | L  [Due to completeness of data was adequate] | U  [No study protocol] | None |
| HuangWF2014[26] | L  [Random number table] | L  [Central random allocation] | L  [Due to use blinding of participants and personnel] | L  [Outcome appraisers implemented a blinding method] | L  [Due to completeness of data was adequate] | U  [Unable to determine prespecified outcomes] | None |
| Shen JF2014[27] | U  [Due to not reported more details] | U  [Not stated] | U  [Not stated] | U  [Not stated] | L  [Due to completeness of data was adequate] | U  [No study protocol] | None |
| Zhao J2015 [28] | U  [Due to not reported more details] | U  [Not stated] | U  [Not stated] | U  [Not stated] | L  [Due to completeness of data was adequate] | U  [No study protocol] | None |
| Wang XS2015[29] | L  [Random number table] | U  [Not stated] | U  [Not stated] | U  [Not stated] | L  [Due to completeness of data was adequate] | U  [No study protocol] | None |
| ChenYL2016[30] | U  [Due to not reported more details] | U  [Not stated] | U  [Not stated] | U  [Not stated] | L  [Due to completeness of data was adequate] | U  [No study protocol] | None |
| Xu CQ2017[31] | H  [Patient's intention] | U  [Not stated] | H  [Due to no blinding] | U  [Not stated] | L  [Due to completeness of data was adequate] | U  [No study protocol] | None |
| Liu R2018 [32] | U  [Due to not reported more details] | U  [Not stated] | U  [Not stated] | U  [Not stated] | L  [Due to completeness of data was adequate] | U  [No study protocol] | None |
| Wen LJ 2018[33] | U  [Due to not reported more details] | U  [Not stated] | U  [Not stated] | U  [Not stated] | L  [Due to completeness of data was adequate] | U  [No study protocol] | None |
| Xia N 2018[34] | U  [Due to not reported more details] | U  [Not stated] | U  [Not stated] | U  [Not stated] | L  [Due to completeness of data was adequate] | U  [No study protocol] | None |

**Abbreviations:** L: low risk of bias; H: high risk of bias; U: unclear risk of bias
