# Supplementary material for: Detoxification therapy of traditional Chinese medicine for genital tract high-risk human papillomavirus infection: A systematic review and meta-analysis
Source: PLoS One. 2019 Mar 1;14(3):e0213062. doi: 10.1371/journal.pone.0213062 (PMC6396931; doi:10.1371/journal.pone.0213062)
Supplement: S3 Table — (DOCX) [file pone.0213062.s003.docx]

**S3 Table. Summary of findings**

| **Intervention** | **Comparison** | **Outcomes** | **Relative effect**  **(95% CI)** | **Standardized mean difference (95% CI)** | **No of participants**  **(Trials)** | **Quality of the evidence**  **(GRADE)** |
| --- | --- | --- | --- | --- | --- | --- |
| DTCM treatment | Follow-up | Rate of hr-HPV clearance | RR 1.88  (1.60 to 2.22) | / | 1709  (14 trials) | low |
|  |  | Regression rate of CIN | RR 1.79  (1.31 to 2.45) | / | 674  (5 trials) | very low |
|  |  | The impact on the level of TNF-α | / | MD 2.99  (1.90 to 4.07) | 140  (2 trials) | very low |
|  |  | The impact on the level of IFN-α | / | MD 3.47  (2.42 to 4.52) | 140  (2 trials) | very low |
|  |  | The impact on the proportion of CD4+/CD8+ cells | / | MD 0.21  (0.05 to 0.37) | 65  (1trial) | very low |
| DTCM treatment | Placebo | Rate of hr-HPV clearance | RR 2.62  (1.28 to 5.33) | / | 197  (3 trials) | very low |
|  |  | Regression rate of CIN | RR 3.61  (1.21 to 10.83) | / | 150  (2 trials) | very low |
