# Supplementary material for: Detoxification therapy of traditional Chinese medicine for genital tract high-risk human papillomavirus infection: A systematic review and meta-analysis
Source: PLoS One. 2019 Mar 1;14(3):e0213062. doi: 10.1371/journal.pone.0213062 (PMC6396931; doi:10.1371/journal.pone.0213062)
Supplement: S1 File — (DOCX) [file pone.0213062.s004.docx]

**S1 File. Electronic search strategy**

Draft MEDLINE search - Ovid interface:

1. Alternative medicine /

2. Plants, Medicinal/

3. medicine, oriental traditional/

4. Medicine, East Asian Traditional/

5. Drugs, Chinese Herbs/

6. [Chinese patent medicine](https://en.wikipedia.org/wiki/Chinese_patent_medicine)/

7. (alternat*adj2 medicine).mp.

8. or/1-7

9. exp human papillomavirus infections /

10. HPV.mp

11. ASC-US.mp

12. LSIL.mp.

13. or/9-12

14. Randomized controlled trail/

15. Controlled clinical trial/

16. (Randomi*adj2 control).mp

17. Trial/

18. or/14-17

19. 8 and 13 and 18

Take CNKI as an example. The Chinese search strategy is as follows:

(SU%HPV or SU% hr-HPV感染 or SU%带下病or SU% 高危型HPV感染)

and (SU%中医 or SU%中西医 or SU%中医疗法or SU%替代医学or SU%草药 or SU%中草药 or SU%中药 or SU%中药疗法 or SU%中成药 or SU%植物药 or SU%中医治法)

and (SU%临床观察 or SU%临床评估 or SU%临床试验 or SU%临床效果 or SU%临床研究 or SU%疗效 or SU%前瞻性 or SU%随访 or SU%多中心 or SU%随机 or SU%对照)
