# Supplementary material for: Detoxification therapy of traditional Chinese medicine for genital tract high-risk human papillomavirus infection: A systematic review and meta-analysis
Source: PLoS One. 2019 Mar 1;14(3):e0213062. doi: 10.1371/journal.pone.0213062 (PMC6396931; doi:10.1371/journal.pone.0213062)
Supplement: S2 File — (DOCX) [file pone.0213062.s005.docx]

**S2 File. Minimal underlying data of all included studies**

| 1.Lou JY 2011 | | |
| --- | --- | --- |
| **Methods** | Randomized controlled trial. | |
| **Participants** | **Inclusion criteria:** women admitted to Beijing University of Traditional Chinese Medicine (TCM) Dong fang Hospital from August 2009 to January 2011 with the diagnosis of hr-HVP. Diagnosis of persistent HPV was based on *Screening and Early Diagnosis and Treatment of Cervical Cancer Guideline*, *Guide to Cervical Cytology Screening*, and *Guidelines for Screening and Handling Cervical Lesions*. **Exclusion criteria:** 1. Patients with other genital tract inflammation: such as gonorrhea, mycoplasma or chlamydia infection and Candida vaginitis, trichomonas vaginitis, bacterial vaginosis, etc.; 2. Cervical disease in CIN II+; 3. Pregnancy or breastfeeding Women in the period; 4. Patients with low immune function. **Number of women randomized:** 60  **Number of women analyzed:** group A: 30; group B: 30. **Number of withdrawals/exclusions/loss to follow-up and reasons:** none **Number of centers:** 1. **Age (years):** group A: 32.23±7.24; group B: 32.56±7.18  **Screening/ Diagnostic Methods:** Hybrid capture-2(HC-2); Thin Prep cytology test (TCT).  **Type of hr-HPV (population size):** no detailed information **Country:** China. | |
| **Interventions** | **Group A:** Qingdu Vaginal Suppository (ingredients include: Pharmaceutical ingredients: sputum, comfrey, sputum, etc.), 570mg. Suppository was placed in the posterior sac of the vagina, once every other day, used before going to bed. Treatment continued for 10 days as a course of treatment, and a total of 3 courses were used. The medication was stopped during the menstrual period. It is forbidden to take a bath and sexual intercourse during the medication.  **Group B:** Just follow-up | |
| **Outcomes** | **Primary outcome:**  Hr-HPV clearance.HC-2 virus ratio＜1 pg/ml  Number of events: within 6 months group A:11; group B:5  **Secondary outcome:**  Adverse events related to TCM detoxification therapy.  Number of events: group A:0; group B:0.  **Each of time of the study visits after the end of treatment(months):**3 months | |
| **Notes** |  | |
| Risk of bias |  |  |
| **Bias** | **Authors’ judgment** | **Support for judgment** |
| Random sequence generation (selection bias) | Unclear risk | Not reported more random sequence generated details. Women randomly assigned to receive Qingdu Vaginal Suppository treatment or follow-up. |
| Allocation concealment (selection bias) | Unclear risk | Not stated. |
| Blinding of participants and personnel (performance bias) All outcomes | High risk | Due to no blinding. |
| Blinding of outcome assessment (detection bias) All outcomes | Unclear risk | Not stated. |
| Incomplete outcome data (attrition bias) All outcomes | Low risk | All women randomized were analyzed. |
| Selective reporting (reporting bias) | Unclear risk | Unable to determine prespecified outcomes. |
| Other bias | None |  |

| 2.Xiao J2011 | | |
| --- | --- | --- |
| **Methods** | Randomized controlled trial. | |
| **Participants** | **Inclusion criteria:** women admitted to Guangdong Provincial Hospital of TCM from October 2005 to February 2008 with the diagnosis of hr-HVP. Diagnosis of persistent HPV was based on *"Medical Science and Technology" textbook,* and *Guide to Cervical Cytology Screening*, and *Guidelines for Screening and Handling Cervical Lesions*. **Exclusion criteria:** 1. Patients with other genital tract inflammation: such as gonorrhea, mycoplasma or chlamydia infection and Candida vaginitis, trichomonas vaginitis, bacterial vaginosis, etc.; 2. Cervical disease in CIN I+. **Number of women randomized:** 47/70  **Number of women analyzed:** group A: 36; group B: 11. **Number of withdrawals/exclusions/loss to follow-up and reasons:**23, due to some patients refused to provide medical history data and cooperate with review. **Number of centers:** 1. **Age (years):** group A: 33.08±7.73;group B:34.18±7.86  **Screening/ Diagnostic Methods:** Hybrid capture-2; Thin Prep cytology test.  **Type of hr-HPV (population size):** no detailed information **Country:** China. | |
| **Interventions** | **Group A:** Youdujing external lotion (Ingredients include: comfrey, Polygonum cuspidatum, Daqingye, Banlangen, Sophora flavescens, Phellodendron, etc.),100ml, vaginal irrigation, twice a week; Youdujing cream (Ingredients include Brucea, comfrey, scorpion, alum, asarum, white, etc.), applying on the cervix, twice a week. Treatment continued for 3 weeks as a course of treatment, and a total of 3 courses are used. The medication was stopped during the menstrual period. It was forbidden to take a bath and sexual intercourse during the medication.  **Group B:** 0.9% physiological saline solution, vaginal irrigation, and applying on the cervix, twice a week. Treatment continued for 3 weeks as a course of treatment, and a total of 3 courses are used. The medication was stopped during the menstrual period. | |
| **Outcomes** | **Primary outcome:**   1. hr-HPV clearance: HPV testing was negative.   Number of events: within 3 months group A:15; group B:2  At 6 months group A:20; group B:4.  (2) Reversal rate of cervical CIN at 6 months: Cytology test throw ThinPrep cytology test (TCT)  Number of events: group A:20; group B:3.  **Each of time of the study visits after the end of treatment(months):** 3,6 months | |
| **Notes** |  | |
| Risk of bias |  |  |
| **Bias** | **Authors’ judgment** | **Support for judgment** |
| Random sequence generation (selection bias) | Unclear risk | Not reported more random sequence generated details. |
| Allocation concealment (selection bias) | Unclear risk | Not stated. |
| Blinding of participants and personnel (performance bias) All outcomes | Low risk | Due to use blinding of participants. |
| Blinding of outcome assessment (detection bias) All outcomes | Unclear risk | Not stated. |
| Incomplete outcome data (attrition bias) All outcomes | High risk | 32% (23/70) of data missing. |
| Selective reporting (reporting bias) | Unclear risk | No study protocol, unable to determine prespecified outcomes. |
| Other bias | None |  |

| 3.Yan X2012 | | |
| --- | --- | --- |
| **Methods** | Randomized controlled trial. | |
| **Participants** | **Inclusion criteria:** women admitted to Guangdong Provincial Hospital of TCM from May 2010 to March 2012 with the diagnosis of hr-HVP. Diagnosis of persistent HPV was based on *Screening and Early Diagnosis and Treatment of Cervical Cancer Guideline*, *Guide to Cervical Cytology Screening*. **Exclusion criteria:** 1. Patients with other genital tract inflammation: such as gonorrhea, mycoplasma or chlamydia infection and Candida vaginitis, trichomonas vaginitis, bacterial vaginosis, etc.; 2. Pregnancy or breastfeeding Women in the period; 3. Patients with severe liver or kidney disease. **Number of women randomized:** 65  **Number of women analyzed:** group A: 32; group B: 33. **Number of withdrawals/exclusions/loss to follow-up and reasons:** none **Number of centers:** 1. **Age (years):** group A: 37.97±9.10;group B:34.94±9.14  **Screening/ Diagnostic Methods:** Polymerase chain reaction (PCR); Thin Prep cytology test. Colposcope  **Type of hr-HPV (population size):** no detailed information **Country:** China. | |
| **Interventions** | **Group A:** Zhidai tablet (ingredients include: Atractylodes, Chinese yam, Alisma, Psyllium, Chuanxiong, Houttuynia and Angelica, et al.), oral,12 pills/d. Treatment continued for 3 months. The medication was stopped during the menstrual period. **Group B:** Just follow-up | |
| **Outcomes** | **Primary outcome:**  Hr-HPV clearance. HPV testing was negative.  Number of events: witinh 6 months group A:14; group B:8  At 12 months group A:17; group B:12.  **Secondary outcome:**  Proportion of immune cells. Detection by enzyme-linked immunosorbent assay (ELISA), the operation is carried out according to the kit instructions  Continuous variables: within 3 months TNF-α: group A:9.3±3.93; group B:6.15±3.36. IFN-α: group A:8.96±4.02; group B:5.35±4.35.  with 6 months TNF-α: group A:9.68±4.62; group B:7.32±4.95. IFN-α: group A:8.34±4.18; group B:4.53±2.8.  **Every of time of the study visits after the end of treatment(months):**3,8 months | |
| **Notes** |  | |
| Risk of bias |  |  |
| **Bias** | **Authors’ judgment** | **Support for judgment** |
| Random sequence generation (selection bias) | Unclear risk | Not reported more random sequence generated details. |
| Allocation concealment (selection bias) | Unclear risk | Not stated. |
| Blinding of participants and personnel (performance bias) All outcomes | Unclear risk | Not stated. |
| Blinding of outcome assessment (detection bias) All outcomes | Unclear risk | Not stated. |
| Incomplete outcome data (attrition bias) All outcomes | Low risk | All women randomized were analyzed. |
| Selective reporting (reporting bias) | Unclear risk | No study protocol, unable to determine prespecified outcomes. |
| Other bias | None |  |

| 4.Xiao J2012 | | |
| --- | --- | --- |
| **Methods** | Randomized controlled trial. | |
| **Participants** | **Inclusion criteria:** women admitted to Guangdong Provincial Hospital of TCM from March 2010 to December 2010 with the diagnosis of hr-HVP. Diagnosis of persistent HPV was based on *Screening and Early Diagnosis and Treatment of Cervical Cancer Guideline*, *Guide to Cervical Cytology Screening*. **Exclusion criteria:** 1. Patients with other genital tract inflammation: such as gonorrhea, mycoplasma or chlamydia infection and Candida vaginitis, trichomonas vaginitis, bacterial vaginosis, etc.; 2. Pregnancy or breastfeeding Women in the period; 3. Patients with severe liver or kidney disease. **Number of women randomized: 70**  **Number of women analyzed:** group A: 35; group B: 35. **Number of withdrawals/exclusions/loss to follow-up and reasons:** 11,due to the patient refused to return to the hospital for review. **Number of centers:** 1. **Age (years):** group A: 34.20±7.90;group B:33.0±7.0  **Screening/ Diagnostic Methods:** Polymerase chain reaction (PCR); Thin Prep cytology test. Colposcope  **Type of hr-HPV (population size):** no detailed information **Country:** China. | |
| **Interventions** | **Group A:** Youdujing external lotion (Ingredients include: comfrey, Polygonum cuspidatum, Daqingye, Banlangen, Sophora flavescens, Phellodendron, etc.),100ml, vaginal irrigation, twice a week; Youdujing cream (Ingredients include Brucea, comfrey, scorpion, alum, asarum, white, etc.), applying on the cervix, twice a week. Treatment continued for 3 weeks as a course of treatment, and a total of 3 courses are used. The medication was stopped during the menstrual period. It was forbidden to take a bath and sexual intercourse during the medication.  **Group B:** 0.9% physiological saline solution, vaginal irrigation, and applying on the cervix, twice a week. Treatment continued for 3 weeks as a course of treatment, and a total of 3 courses are used. The medication was stopped during the menstrual period. | |
| **Outcomes** | **Primary outcome:**   1. hr-HPV clearance: HPV testing was negative.   Number of events: within 3 months group A:18; group B:5.  2) Reversal rate of cervical CIN: Cytology test throw ThinPrep cytology test (TCT)  Number of events: within 3 months group A:9; group B:2.  **Every of time of the study visits after the end of treatment(months):** 3 months | |
| **Notes** |  | |
| Risk of bias |  |  |
| **Bias** | **Authors’ judgment** | **Support for judgment** |
| Random sequence generation (selection bias) | Unclear risk | Not reported more random sequence generated details. |
| Allocation concealment (selection bias) | Unclear risk | Not stated. |
| Blinding of participants and personnel (performance bias) All outcomes | Low risk | Due to use blinding of participants. |
| Blinding of outcome assessment (detection bias) All outcomes | Unclear risk | Not stated. |
| Incomplete outcome data (attrition bias) All outcomes | Low risk | Due to15% (11/70) of data missing. |
| Selective reporting (reporting bias) | Unclear risk | No study protocol, unable to determine prespecified outcomes. |
| Other bias | None |  |

| 5.Zhang J2012 | | |
| --- | --- | --- |
| **Methods** | Randomized controlled trial. | |
| **Participants** | **Inclusion criteria:** women admitted to Guangdong Provincial Hospital of TCM from January 2010 to September 2011 with the diagnosis of hr-HVP. Diagnosis of persistent HPV was based on *Screening and Early Diagnosis and Treatment of Cervical Cancer Guideline*, *Guide to Cervical Cytology Screening*. **Exclusion criteria: 1.**Age less than 18 years old or older than 60 years old; 2.pregnancy Or lactating women; 3.combined with severe primary features such as cardiovascular, hepatic, renal and hematopoietic systems People with diseases, mental illnesses, and malignant tumors; 4.those who are allergic to research drugs. **Number of women randomized:** 75  **Number of women analyzed:** group A: 41; group B: 34. **Number of withdrawals/exclusions/loss to follow-up and reasons:** none **Number of centers:** 1. **Age (years):** group A: 35.98±8.97;group B:34.33±9.58  **Screening/ Diagnostic Methods:** Polymerase chain reaction (PCR); Thin Prep cytology test. Colposcope  **Type of hr-HPV (population size):** no detailed information **Country:** China. | |
| **Interventions** | **Group A:** Zhidai tablet (ingredients include: Atractylodes, Chinese yam, Alisma, Psyllium, Chuanxiong, Houttuynia and Angelica, et al.), oral,12 pills/d. Treatment continued for 3 months. The medication was stopped during the menstrual period. **Group B:** Just follow-up | |
| **Outcomes** | **Primary outcome:**  Hr-HPV clearance. HPV testing was negative.  Number of events: within 6 months group A:18; group B:7.  **Secondary outcome:**  Proportion of immune cells. Detection by enzyme-linked immunosorbent assay (ELISA), the operation is carried out according to the kit instructions  Continuous variables: within 3 months TNF-α: group A:9.26±4.04; group B:6.09±3.42. IFN-α: group A:8.76±4.32; group B:5.73±3.33.  **Every of time of the study visits after the end of treatment(months):**3 months | |
| **Notes** |  | |
| Risk of bias |  |  |
| **Bias** | **Authors’ judgment** | **Support for judgment** |
| Random sequence generation (selection bias) | Unclear risk | Not reported more random sequence generated details. |
| Allocation concealment (selection bias) | Unclear risk | Not stated. |
| Blinding of participants and personnel (performance bias) All outcomes | Unclear risk | Not stated. |
| Blinding of outcome assessment (detection bias) All outcomes | Unclear risk | Not stated. |
| Incomplete outcome data (attrition bias) All outcomes | Low risk | All women randomized were analyzed. |
| Selective reporting (reporting bias) | Unclear risk | No study protocol, unable to determine prespecified outcomes. |
| Other bias | None |  |

| 6.Shen JJ 2013 | | |
| --- | --- | --- |
| **Methods** | Randomized controlled trial. | |
| **Participants** | **Inclusion criteria:** women admitted to Beijing University of Shenzhen Hospital from July 2007 to March 2011 with the diagnosis of hr-HVP. Diagnosis of persistent HPV was based on *Screening and Early Diagnosis and Treatment of Cervical Cancer Guideline*. **Exclusion criteria:** 1. Patients with other genital tract inflammation: such as gonorrhea, mycoplasma or chlamydia infection and Candida vaginitis, trichomonas vaginitis, bacterial vaginosis, etc.; 2. Pregnancy or breastfeeding Women in the period; 3. Patients with severe liver or kidney disease. **Number of women randomized: 226**  **Number of women analyzed:** group A: 113; group B: 113. **Number of withdrawals/exclusions/loss to follow-up and reasons:** 38,due to the patient refused to return to the hospital for review. **Number of centers:** 1. **Age (years):** group A: 31.78±6.54;group B:3 3.59±7.41  **Screening/ Diagnostic Methods:** HC-2; Thin Prep cytology test. Colposcope  **Type of hr-HPV (population size):** no detailed information **Country:** China. | |
| **Interventions** | **Group A:** Baofukang Suppository (Ingredients include: zedoary oil and borneol),3.48g, Suppository was placed in the posterior sac of the vagina, once every day, used before going to bed. Treatment continued for 16 days as a course of treatment, and a total of 3 courses are used. The medication was stopped during the menstrual period. It was forbidden to take a bath and sexual intercourse during the medication.  **Group B:** Just follow-up. | |
| **Outcomes** | **Primary outcome:**  1.hr-HPV clearance: HPV testing was negative.  Number of events: within 6 months group A:31; group B:26.  at 12 months group A:66; group B:43.  at 24 months group A:73; group B:54.  2.Reversal rate of cervical CIN: Cytology test throw ThinPrep cytology test (TCT)  Number of events: within 6 months group A:30; group B:26  at 24 months group A:60; group B:54.  **Every of time of the study visits after the end of treatment(months):** 6,12,24 months | |
| **Notes** |  | |
| Risk of bias |  |  |
| **Bias** | **Authors’ judgment** | **Support for judgment** |
| Random sequence generation (selection bias) | Unclear risk | Not reported more random sequence generated details. |
| Allocation concealment (selection bias) | Unclear risk | Not stated. |
| Blinding of participants and personnel (performance bias) All outcomes | Unclear risk | Not stated. |
| Blinding of outcome assessment (detection bias) All outcomes | Unclear risk | Not stated. |
| Incomplete outcome data (attrition bias) All outcomes | Low risk | Due to16.8% (38/226) of data missing. |
| Selective reporting (reporting bias) | Unclear risk | No study protocol, unable to determine prespecified outcomes. |
| Other bias | None |  |

| 7.Xu YX 2013 | | |
| --- | --- | --- |
| **Methods** | Randomized controlled trial. | |
| **Participants** | **Inclusion criteria:** women admitted to Liuzhou People's Hospital from August 2011 to February 2012 with the diagnosis of CIN I with hr-HVP infection. Diagnosis of persistent HPV was based on *Screening and Early Diagnosis and Treatment of Cervical Cancer Guideline*. **Exclusion criteria:** 1. Patients with other genital tract inflammation: such as gonorrhea, mycoplasma or chlamydia infection and Candida vaginitis, trichomonas vaginitis, bacterial vaginosis, etc.; 2. Pregnancy or breastfeeding Women in the period; 3. Patients with severe liver or kidney disease. **Number of women randomized: 100**  **Number of women analyzed:** group A: 50; group B: 50. **Number of withdrawals/exclusions/loss to follow-up and reasons:** 12,due to the patient refused to return to the hospital for review. **Number of centers:** 1. **Age (years):** 25-53  **Screening/ Diagnostic Methods:** PCR, plus Gene-chip method; Thin Prep cytology test. Colposcope  **Type of hr-HPV (population size):** Hr-HPV in combination with low-risk HPV  **Country:** China. | |
| **Interventions** | **Group A:** Erhuang Vaginal Powder, Suppository was placed in the posterior sac of the vagina, once every other day. Treatment continued for 7 days as a course of treatment, and a total of 3 courses are used. The medication was stopped during the menstrual period. It was forbidden to take a bath and sexual intercourse during the medication.  **Group B:** Just follow-up. | |
| **Outcomes** | **Primary outcome:**  1.hr-HPV clearance: HPV testing was negative.  Number of events: within 6 months group A:25; group B:7  at 12 months group A:29; group B:8  2.Reversal rate of cervical CIN: Cytology test throw ThinPrep cytology test (TCT)  Number of events: within 6 months group A:39; group B:15  at 12 months group A:41; group B:16.  **Secondary outcome:**  Adverse events related to DTCM  genital mucosal irritation symptoms. Number of events: group A:5; group B:0.  **Every of time of the study visits after the end of treatment(months):** 3,6,9 months | |
| **Notes** |  | |
| Risk of bias |  |  |
| **Bias** | **Authors’ judgment** | **Support for judgment** |
| Random sequence generation (selection bias) | Unclear risk | Not reported more random sequence generated details. |
| Allocation concealment (selection bias) | Unclear risk | Not stated. |
| Blinding of participants and personnel (performance bias) All outcomes | Unclear risk | Not stated. |
| Blinding of outcome assessment (detection bias) All outcomes | Unclear risk | Not stated. |
| Incomplete outcome data (attrition bias) All outcomes | Low risk | Due to12% (12/100) of data missing |
| Selective reporting (reporting bias) | Unclear risk | No study protocol, unable to determine prespecified outcomes. |
| Other bias | None |  |

| 8.Zhang H2013 | | |
| --- | --- | --- |
| **Methods** | Randomized controlled trial. | |
| **Participants** | **Inclusion criteria:** women admitted to Gaomi City People's Hospital of Shandong Province from October 2010 to September 2012 with the diagnosis of hr-HVP infection. Diagnosis of persistent HPV was based on *Screening and Early Diagnosis and Treatment of Cervical Cancer Guideline*. **Exclusion criteria:** 1. Patients with other genital tract inflammation: such as gonorrhea, mycoplasma or chlamydia infection and Candida vaginitis, trichomonas vaginitis, bacterial vaginosis, etc.; 2. Pregnancy or breastfeeding Women in the period; 3. Patients with severe liver or kidney disease. **Number of women randomized: 110**  **Number of women analyzed:** group A: 70; group B: 40. **Number of withdrawals/exclusions/loss to follow-up and reasons:** none  **Number of centers:** 1. **Age (years):** group A**:**30.00±7.00; group B:31.40±4.98  **Screening/ Diagnostic Methods:** HC-2; Thin Prep cytology test.  **Type of hr-HPV (population size):** none  **Country:** China. | |
| **Interventions** | **Group A:** BaofukangSuppository,3.48g; Suppository was placed in the posterior sac of the vagina, once every other day. +Gubenjiedu Decoction (The drug composition contains: Astragalus 30g, Codonopsis 20g, Angelica 15g, Radix Paeoniae 15g, Radix Isatidis 15g, Lithospermum 15g, Hedyotis diffusa 20g, Guanzhong 12g, Daqingye 15g, Cork 15g), 100ml, oral, bid. Treatment continued for 16 days as a course of treatment, and a total of 3 courses are used. The medication was stopped during the menstrual period. It was forbidden to take a bath and sexual intercourse during the medication.  **Group B:** Just follow-up. | |
| **Outcomes** | **Primary outcome:**  hr-HPV clearance: HPV testing was negative.  Number of events: within 6 months group A:48; group B:13.  **Secondary outcome:**  Adverse events related to DTCM  Number of events: group A:0; group B:0.  **Every of time of the study visits after the end of treatment(months):** 6 months | |
| **Notes** |  | |
| Risk of bias |  |  |
| **Bias** | **Authors’ judgment** | **Support for judgment** |
| Random sequence generation (selection bias) | Unclear risk | Not reported more random sequence generated details. |
| Allocation concealment (selection bias) | Unclear risk | Not stated. |
| Blinding of participants and personnel (performance bias) All outcomes | Unclear risk | Not stated. |
| Blinding of outcome assessment (detection bias) All outcomes | Unclear risk | Not stated. |
| Incomplete outcome data (attrition bias) All outcomes | Low risk | Due to completeness of data was adequate |
| Selective reporting (reporting bias) | Unclear risk | No study protocol, unable to determine prespecified outcomes. |
| Other bias | None |  |

| 9.Huang WF2014 | | |
| --- | --- | --- |
| **Methods** | Randomized controlled trial. | |
| **Participants** | **Inclusion criteria:** women admitted to Affiliated to Guangzhou University of Traditional Chinese Medicine, Shenzhen Chinese Medicine Hospital from December 2011 to December 2013 with the diagnosis of hr-HVP. Diagnosis of persistent HPV was based on *Screening and Early Diagnosis and Treatment of Cervical Cancer Guideline*, *Guide to Cervical Cytology Screening*. **Exclusion criteria:** 1. Patients with other genital tract inflammation: such as gonorrhea, mycoplasma or chlamydia infection and Candida vaginitis, trichomonas vaginitis, bacterial vaginosis, etc.; 2. Pregnancy or breastfeeding Women in the period; 3. Patients with urinary tract obstruction; 4.severe columnar epithelial migration; 5.cervical cancer or cervical intraepithelial neoplasia. **Number of women randomized: 80**  **Number of women analyzed:** group A: 50; group B: 30. **Number of withdrawals/exclusions/loss to follow-up and reasons:** none. **Number of centers:** 1. **Age (years):** 25-56  **Screening/ Diagnostic Methods:** HC-2; Thin Prep cytology test.  **Type of hr-HPV (population size):** no detailed information **Country:** China. | |
| **Interventions** | **Group A:** Gongjingkang Vaginal gel (Ingredients include: Sophora flavescens, Winged, White and, Scorpion oil, borneol, Astragalus, etc.),1.0g, intravaginal administration, once every day. Treatment continued for 2 weeks as a course of treatment, and a total of 3 courses are used. The medication was stopped during the menstrual period. It was forbidden to take a bath and sexual intercourse during the medication.  **Group B:** Placebo gel. ,1.0g, intravaginal administration, once every day. Treatment continued for 3 weeks as a course of treatment, and a total of 3 courses are used. The medication was stopped during the menstrual period. | |
| **Outcomes** | **Primary outcome:**  hr-HPV clearance: HPV testing was negative.  Number of events: within 3 months group A:14; group B:0.  **Secondary outcome:**  Adverse events related to DTCM  Number of events: group A:0; group B:0.  Rate of reoccurrence.  Number of events: at 12 months group A:7; group B:13.  **Every of time of the study visits after the end of treatment(months):** 3,12 months | |
| **Notes** |  | |
| Risk of bias |  |  |
| **Bias** | **Authors’ judgment** | **Support for judgment** |
| Random sequence generation (selection bias) | Low risk | Random number table. |
| Allocation concealment (selection bias) | Low risk | Central random allocation. |
| Blinding of participants and personnel (performance bias) All outcomes | Low risk | Use blinding of participants and personnel. |
| Blinding of outcome assessment (detection bias) All outcomes | Low risk | Outcome appraisers implemented a blinding method. |
| Incomplete outcome data (attrition bias) All outcomes | Low risk | Due to completeness of data was adequate. |
| Selective reporting (reporting bias) | Unclear risk | No study protocol, unable to determine prespecified outcomes. |
| Other bias | None |  |

| 10.Shen JF 2014 | | |
| --- | --- | --- |
| **Methods** | Randomized controlled trial. | |
| **Participants** | **Inclusion criteria:** women admitted to Zhejiang Cixi Maternal and Child Health Hospital from January 2009 to December 2013 with the diagnosis of hr-HVP infection. Diagnosis of persistent HPV was based on *Screening and Early Diagnosis and Treatment of Cervical Cancer Guideline*. **Exclusion criteria:** 1. Patients< 18 years old or > 60 years old; 2.Patients with other genital tract inflammation: such as gonorrhea, mycoplasma or chlamydia infection and Candida vaginitis, trichomonas vaginitis, bacterial vaginosis, etc.; 3. Pregnancy or breastfeeding Women in the period; 4. Patients with physical allergies or allergies to BaofuKang Suppository.  **Number of women randomized: 106**  **Number of women analyzed:** group A: 53; group B: 53. **Number of withdrawals/exclusions/loss to follow-up and reasons:** none  **Number of centers:** 1. **Age (years):** group A**:**46.00±1.00; group B:45.50±0.5  **Screening/ Diagnostic Methods:** HC-2; Thin Prep cytology test; Colposcope.  **Type of hr-HPV (population size):** none  **Country:** China. | |
| **Interventions** | **Group A:** BaofukangSuppository,1.74g; Suppository was placed in the posterior sac of the vagina, once every day. Treatment continued for 14 days as a course of treatment, and a total of 3 courses are used. The medication was stopped during the menstrual period. It was forbidden to take a bath and sexual intercourse during the medication.  **Group B:** Just follow-up. | |
| **Outcomes** | **Primary outcome:**  hr-HPV clearance: HPV testing was negative.  Number of events: within 6 months group A:20; group B:11.  **Every of time of the study visits after the end of treatment(months):** 6 months | |
| **Notes** |  | |
| Risk of bias |  |  |
| **Bias** | **Authors’ judgment** | **Support for judgment** |
| Random sequence generation (selection bias) | Unclear risk | Not reported more random sequence generated details. |
| Allocation concealment (selection bias) | Unclear risk | Not stated. |
| Blinding of participants and personnel (performance bias) All outcomes | Unclear risk | Not stated. |
| Blinding of outcome assessment (detection bias) All outcomes | Unclear risk | Not stated. |
| Incomplete outcome data (attrition bias) All outcomes | Low risk | Due to completeness of data was adequate |
| Selective reporting (reporting bias) | Unclear risk | No study protocol, unable to determine prespecified outcomes. |
| Other bias | None |  |

| 11.Zhao J2015 | | |
| --- | --- | --- |
| **Methods** | Randomized controlled trial. | |
| **Participants** | **Inclusion criteria:** women admitted to Beijing University First Hospital, Shenzhen People's Hospital of Guangdong Province, Renji Hospital affiliated to Shanghai Jiaotong University, Beijing Hospital of Ministry of Health, Tongji Hospital affiliated to Wuhan Tongji Medical University, and Obstetrics and Gynecology Hospital affiliated to Zhejiang University from February 2007 to April 2008 with the diagnosis of hr-HVP infection. Diagnosis of persistent HPV was based on *Screening and Early Diagnosis and Treatment of Cervical Cancer Guideline*. **Exclusion criteria:** 1. Patients with immune dysfunction (after chemotherapy and radiotherapy, HIV, systemic lupus erythematosus, etc.); 2.pregnant, lactating women; 3.acute genital tract inflammation (gonorrhea, mycoplasma or chlamydia infection, etc.); 4.patients who do not receive full treatment and follow-up; 5.Patients who do not use contraceptives during sexual intercourse. **Number of women randomized: 243**  **Number of women analyzed:** group A: 161; group B: 82. **Number of withdrawals/exclusions/loss to follow-up and reasons:** none  **Number of centers:** 6. **Age (years):** group A**:**37.03±9.33; group B:35.82±9.31  **Screening/ Diagnostic Methods:** PCR+ Gene-chip method; Thin Prep cytology test.  **Type of hr-HPV (population size):** HPV16:53; HPV18:19; Other types:171  **Country:** China. | |
| **Interventions** | **Group A:** BaofukangSuppository,3.48g; Suppository was placed in the posterior sac of the vagina, once every day. Treatment continued for 16 days as a course of treatment, and a total of 3 courses are used. The medication was stopped during the menstrual period. It was forbidden to take a bath and sexual intercourse during the medication.  **Group B:** Just follow-up. | |
| **Outcomes** | **Primary outcome:**  hr-HPV clearance: HPV testing was negative.  Number of events: within 6 months group A:96; group B:21  at 12 months group A:112; group B:32.  **Secondary outcome:**  Adverse events related to DTCM  Number of events: group A:0; group B:0.  **Every of time of the study visits after the end of treatment(months):** 4,8 months | |
| **Notes** |  | |
| Risk of bias |  |  |
| **Bias** | **Authors’ judgment** | **Support for judgment** |
| Random sequence generation (selection bias) | Unclear risk | Not reported more random sequence generated details. |
| Allocation concealment (selection bias) | Unclear risk | Not stated. |
| Blinding of participants and personnel (performance bias) All outcomes | Unclear risk | Not stated. |
| Blinding of outcome assessment (detection bias) All outcomes | Unclear risk | Not stated. |
| Incomplete outcome data (attrition bias) All outcomes | Low risk | Due to completeness of data was adequate |
| Selective reporting (reporting bias) | Unclear risk | No study protocol, unable to determine prespecified outcomes. |
| Other bias | None |  |

| 12.Wang XS2015 | | |
| --- | --- | --- |
| **Methods** | Randomized controlled trial. | |
| **Participants** | **Inclusion criteria:** women admitted to Chongqing Hospital of TCM from January 2009 to December 2013 with the diagnosis of hr-HVP infection. Diagnosis of persistent HPV was based on *Screening and Early Diagnosis and Treatment of Cervical Cancer Guideline*. **Exclusion criteria:** 1. Patients <18 years old or >60 years old; 2.patients with genital tract inflammation; 3.patients with cervical precancerous lesions or pregnant or lactating women; 4.patients with mental illness, difficult to obey arrangements; low immunity or allergy to Baofukang suppository; 5.patients For trichomoniasis and mold and chlamydia infection; 6.patients with liver and kidney dysfunction history; 7.patients with history of cardiovascular and cerebrovascular diseases. 8.Non-menopausal women with irregular menstruation and total uterine cuts and sexual intercourse do not use condoms, allergies. **Number of women randomized: 106**  **Number of women analyzed:** group A: 53; group B: 53. **Number of withdrawals/exclusions/loss to follow-up and reasons:** none  **Number of centers:** 1. **Age (years):** group A**:**46.00±1.00; group B:45.5±0.5  **Screening/ Diagnostic Methods:** HC-2; Thin Prep cytology test.  **Type of hr-HPV (population size):** none  **Country:** China. | |
| **Interventions** | **Group A:** BaofukangSuppository,1.74g; Suppository was placed in the posterior sac of the vagina, once every day. Treatment continued for 14 days as a course of treatment, and a total of 3 courses are used. The medication was stopped during the menstrual period. It was forbidden to take a bath and sexual intercourse during the medication.  **Group B:** Just follow-up. | |
| **Outcomes** | **Primary outcome:**  hr-HPV clearance: HPV testing was negative.  Number of events: within 6 months group A:31; group B:10.  **Every of time of the study visits after the end of treatment(months):** 3 months | |
| **Notes** |  | |
| Risk of bias |  |  |
| **Bias** | **Authors’ judgment** | **Support for judgment** |
| Random sequence generation (selection bias) | Low risk | Random number table. |
| Allocation concealment (selection bias) | Unclear risk | Not stated. |
| Blinding of participants and personnel (performance bias) All outcomes | Unclear risk | Not stated. |
| Blinding of outcome assessment (detection bias) All outcomes | Unclear risk | Not stated. |
| Incomplete outcome data (attrition bias) All outcomes | Low risk | Due to completeness of data was adequate |
| Selective reporting (reporting bias) | Unclear risk | No study protocol, unable to determine prespecified outcomes. |
| Other bias | None |  |

| 13.Chen YL2016 | | |
| --- | --- | --- |
| **Methods** | Randomized controlled trial. | |
| **Participants** | **Inclusion criteria:** women admitted to Luliang County People's Hospital, Qujing City, Yunnan Province from February 2011 to February 2015 with the diagnosis of hr-HVP infection. Diagnosis of persistent HPV was based on *Screening and Early Diagnosis and Treatment of Cervical Cancer Guideline*. **Exclusion criteria:** 1. Women during pregnancy, lactation or intentional pregnancy. 2. Patients with a history of total uterine disease. 3. The use of oral contraceptive contraceptives in the past 3 months. 4. Allergic constitution or allergy to Baofukang suppository. **Number of women randomized: 200**  **Number of women analyzed:** group A: 100; group B: 100. **Number of withdrawals/exclusions/loss to follow-up and reasons:** none  **Number of centers:** 1. **Age (years):** 25-55  **Screening/ Diagnostic Methods:** HC-2; Thin Prep cytology test; Colposcope.  **Type of hr-HPV (population size):** none  **Country:** China. | |
| **Interventions** | **Group A:** BaofukangSuppository,1.74g; Suppository was placed in the posterior sac of the vagina, once every day. Treatment continued for 14 days as a course of treatment, and a total of 3 courses are used. The medication was stopped during the menstrual period. It was forbidden to take a bath and sexual intercourse during the medication.  **Group B:** Just follow-up. | |
| **Outcomes** | **Primary outcome:**  hr-HPV clearance: HPV testing was negative.  Number of events: within 6 months group A:43; group B:19.  **Every of time of the study visits after the end of treatment(months):** 3 months | |
| **Notes** |  | |
| Risk of bias |  |  |
| **Bias** | **Authors’ judgment** | **Support for judgment** |
| Random sequence generation (selection bias) | Unclear risk | Not reported more random sequence generated details. |
| Allocation concealment (selection bias) | Unclear risk | Not stated. |
| Blinding of participants and personnel (performance bias) All outcomes | Unclear risk | Not stated. |
| Blinding of outcome assessment (detection bias) All outcomes | Unclear risk | Not stated. |
| Incomplete outcome data (attrition bias) All outcomes | Low risk | Due to completeness of data was adequate |
| Selective reporting (reporting bias) | Unclear risk | No study protocol, unable to determine prespecified outcomes. |
| Other bias | None |  |

| 14.Xu CQ2017 | | |
| --- | --- | --- |
| **Methods** | Randomized controlled trial. | |
| **Participants** | **Inclusion criteria:** women admitted to Qinhuangdao City Maternal and Child Health Hospital, Hebei Province from February 2011 to December 2014 with the diagnosis of hr-HVP infection. Diagnosis of persistent HPV was based on *Screening and Early Diagnosis and Treatment of Cervical Cancer Guideline*. **Exclusion criteria:** 1. Women during pregnancy, lactation or intentional pregnancy. 2. Patients with mental illness, difficult to obey arrangements; low immunity or allergy to Baofukang suppository. 3. The use of oral contraceptive contraceptives in the past 3 months. 4. Allergic constitution or allergy to Baofukang suppository. **Number of women randomized: 251**  **Number of women analyzed:** group A: 130; group B: 121. **Number of withdrawals/exclusions/loss to follow-up and reasons:** none  **Number of centers:** 1. **Age (years):** group A**:**34.9±6.1; group B:35.1±5.9  **Screening/ Diagnostic Methods:** HC-2; Thin Prep cytology test; Colposcope.  **Type of hr-HPV (population size):** none  **Country:** China. | |
| **Interventions** | **Group A:** BaofukangSuppository,3.48g; Suppository was placed in the posterior sac of the vagina, once every day. + Chinese herbal Decoction: Houttuynia 15 g, Astragalus 15 g, Atractylodes 10 g, Codonopsis 10 g, Poria 10 g, licorice 10 g, Coix seed 15 g, 100ml, oral, 3 times a day. Treatment continued for 14 days as a course of treatment, and a total of 3 courses are used. The medication was stopped during the menstrual period. It was forbidden to take a bath and sexual intercourse during the medication.  **Group B:** Just follow-up. | |
| **Outcomes** | **Primary outcome:**  1.hr-HPV clearance: HPV testing was negative.  Number of events: at 12 months group A:49; group B:37.  at 24 months group A:93; group B:68  2.Reversal rate of cervical CIN: Cytology test throw ThinPrep cytology test (TCT)  Number of events: at 12 months group A:49; group B:37.  at 24 months group A:93; group B:68  **Every of time of the study visits after the end of treatment(months):** 12,24 months | |
| **Notes** |  | |
| Risk of bias |  |  |
| **Bias** | **Authors’ judgment** | **Support for judgment** |
| Random sequence generation (selection bias) | High risk | Patient's intention. |
| Allocation concealment (selection bias) | Unclear risk | Not stated. |
| Blinding of participants and personnel (performance bias) All outcomes | High risk | Due to no blinding. |
| Blinding of outcome assessment (detection bias) All outcomes | Unclear risk | Not stated. |
| Incomplete outcome data (attrition bias) All outcomes | Low risk | Due to completeness of data was adequate |
| Selective reporting (reporting bias) | Unclear risk | No study protocol, unable to determine prespecified outcomes. |
| Other bias | None |  |

| 15.Liu R 2018 | | |
| --- | --- | --- |
| **Methods** | Randomized controlled trial. | |
| **Participants** | **Inclusion criteria:** women admitted to Huazhong University of Science Tongji Hospital, Tongji Medical College from Febril 2015 to June 2016 with the diagnosis of hr-HVP infection. Diagnosis of persistent HPV was based on *2012 updated consensus guidelines for the management of abnormal cervical cancer screening tests and cancer precursors*. **Exclusion criteria:** 1. Women during pregnancy, lactation or intentional pregnancy. 2. Patients without a history of other systemic malignancies. 3. Allergic constitution. **Number of women randomized: 40**  **Number of women analyzed:** group A: 20; group B: 20. **Number of withdrawals/exclusions/loss to follow-up and reasons:** none  **Number of centers:** 1. **Age (years):** 18-54(26±3)  **Screening/ Diagnostic Methods:** HC-2; Thin Prep cytology test; Colposcope.  **Type of hr-HPV (population size):** none  **Country:** China. | |
| **Interventions** | **Group A:** Erhuang Vaginal Suppository (Realgar, etc.); Suppository was placed in the posterior sac of the vagina, once every day. Treatment continued for 14 days as a course of treatment, and a total of 3 courses are used. The medication was stopped during the menstrual period. It was forbidden to take a bath and sexual intercourse during the medication.  **Group B:** Just follow-up. | |
| **Outcomes** | **Primary outcome:**  1.hr-HPV clearance: HPV testing was negative.  Number of events: within 6 months group A:17; group B:0.  2.Reversal rate of cervical CIN: Cytology test throw ThinPrep cytology test (TCT)  Number of events: group A:15; group B:0.  **Every of time of the study visits after the end of treatment(months):** 3,9 months | |
| **Notes** |  | |
| Risk of bias |  |  |
| **Bias** | **Authors’ judgment** | **Support for judgment** |
| Random sequence generation (selection bias) | Unclear risk | Not reported more random sequence generated details. |
| Allocation concealment (selection bias) | Unclear risk | Not stated. |
| Blinding of participants and personnel (performance bias) All outcomes | Unclear risk | Not stated. |
| Blinding of outcome assessment (detection bias) All outcomes | Unclear risk | Not stated. |
| Incomplete outcome data (attrition bias) All outcomes | Low risk | Due to completeness of data was adequate |
| Selective reporting (reporting bias) | Unclear risk | No study protocol, unable to determine prespecified outcomes. |
| Other bias | None |  |

| 16.Wen LJ 2018 | | |
| --- | --- | --- |
| **Methods** | Randomized controlled trial. | |
| **Participants** | **Inclusion criteria:** women admitted to Beijing University of TCM Hospital from January 2016 to January 2017 with the diagnosis of hr-HVP infection. Diagnosis of persistent HPV was based on *2012 updated consensus guidelines for the management of abnormal cervical cancer screening tests and cancer precursors*. **Exclusion criteria:** 1. Women during pregnancy, lactation or intentional pregnancy. 2. Allergic constitution or allergy to Baofukang suppository. **Number of women randomized: 70**  **Number of women analyzed:** group A: 35; group B: 35. **Number of withdrawals/exclusions/loss to follow-up and reasons:** none  **Number of centers:** 1. **Age (years):** group A**:**35.4±6.51; group B:34.0±6.23  **Screening/ Diagnostic Methods:** HC-2; Thin Prep cytology test.  **Type of hr-HPV (population size):** none  **Country:** China. | |
| **Interventions** | **Group A:** Chinese compound herb Vaginal Powder (Ingredients include: comfrey, cork, scorpion, borneol, etc.), 3g; Suppository was placed in the posterior sac of the vagina, once every other day. Treatment continued for 10 days as a course of treatment, and a total of 3 courses are used. The medication was stopped during the menstrual period. It was forbidden to take a bath and sexual intercourse during the medication.  **Group B:** Just follow-up. | |
| **Outcomes** | **Primary outcome:**  hr-HPV clearance: HPV testing was negative.  Number of events: within 6 months group A:14; group B:5.  At 12 months group A:19; group B:7.  **Secondary outcome:**  Adverse events related to DTCM  Number of events: group A:0; group B:0.  **Every of time of the study visits after the end of treatment(months):** 4,12 months | |
| **Notes** |  | |
| Risk of bias |  |  |
| **Bias** | **Authors’ judgment** | **Support for judgment** |
| Random sequence generation (selection bias) | Unclear risk | Not reported more random sequence generated details. |
| Allocation concealment (selection bias) | Unclear risk | Not stated. |
| Blinding of participants and personnel (performance bias) All outcomes | Unclear risk | Not stated. |
| Blinding of outcome assessment (detection bias) All outcomes | Unclear risk | Not stated. |
| Incomplete outcome data (attrition bias) All outcomes | Low risk | Due to completeness of data was adequate |
| Selective reporting (reporting bias) | Unclear risk | No study protocol, unable to determine prespecified outcomes. |
| Other bias | None |  |

| 17.Xia N2018 | | |
| --- | --- | --- |
| **Methods** | Randomized controlled trial. | |
| **Participants** | **Inclusion criteria:** women admitted to Jiangsu Provincial Hospital of Traditional Chinese Medicine from January 2017 to August 2017 with the diagnosis of CIN I with hr-HVP infection. Diagnosis of persistent HPV was based on *"Gynecology and Obstetrics" textbook, (2013 edition) and 2012 updated consensus guidelines for the management of abnormal cervical cancer screening tests and cancer precursors*. **Exclusion criteria:** 1. Women during pregnancy, lactation or intentional pregnancy. 2. patients with history of chronic underlying disease or liver and kidney dysfunction affecting medication; 3.patients with history of cervical polyps, history of physical therapy due to cervical lesions, or history of previous cervical trauma; 4.Patients with history of multiple drug food allergies, especially those with Chinese medicine. **Number of women randomized: 57**  **Number of women analyzed:** group A: 29; group B: 28. **Number of withdrawals/exclusions/loss to follow-up and reasons:** none  **Number of centers:** 1. **Age (years):** group A**:**39.55±5.17; group B:39.21±5.45  **Screening/ Diagnostic Methods:** HC-2; Thin Prep cytology test; Colposcope.  **Type of hr-HPV (population size):** none  **Country:** China. | |
| **Interventions** | **Group A:** Ermiao Decoction (Ingredients include:Cork 10g, bran fried atractylodes 10g, bran fried atractylodes 10g, coix seed 30g, chonglou 10g, Hedyotis grass 10g, Radix isatidis 10g, bandit 10g.), oral, bid. Treatment continued for 14 days per months as a course of treatment, and a total of 3 courses are used. The medication was stopped during the menstrual period. It was forbidden to take a bath and unprotected sexual intercourse during the medication.  **Group B:** Just follow-up. | |
| **Outcomes** | **Primary outcome:**  hr-HPV clearance: HPV testing was negative.  Number of events: within 6 months group A:19; group B:6.  2.Reversal rate of cervical CIN: Cytology test throw ThinPrep cytology test (TCT)  Number of events: within 6 months group A:18; group B:5.  **Secondary outcome:**  Proportion of immune cells. Detection by enzyme-linked immunosorbent assay (ELISA), the operation is carried out according to the kit instructions  Continuous variables: *CD^4+^/CD^8+^ cells*: group A:12.0±2.9; group B:0.99±0.31.  Adverse events related to DTCM  Number of events: group A:0; group B:0.  **Every of time of the study visits after the end of treatment(months):** 6 months | |
| **Notes** |  | |
| Risk of bias |  |  |
| **Bias** | **Authors’ judgment** | **Support for judgment** |
| Random sequence generation (selection bias) | Unclear risk | Not reported more random sequence generated details. |
| Allocation concealment (selection bias) | Unclear risk | Not stated. |
| Blinding of participants and personnel (performance bias) All outcomes | Unclear risk | Not stated. |
| Blinding of outcome assessment (detection bias) All outcomes | Unclear risk | Not stated. |
| Incomplete outcome data (attrition bias) All outcomes | Low risk | Due to completeness of data was adequate |
| Selective reporting (reporting bias) | Unclear risk | No study protocol, unable to determine prespecified outcomes. |
| Other bias | None |  |
