# Supplementary figures and images for: Detoxification therapy of traditional Chinese medicine for genital tract high-risk human papillomavirus infection: A systematic review and meta-analysis
Source: PLoS One. 2019 Mar 1;14(3):e0213062. doi: 10.1371/journal.pone.0213062 (PMC6396931; doi:10.1371/journal.pone.0213062)

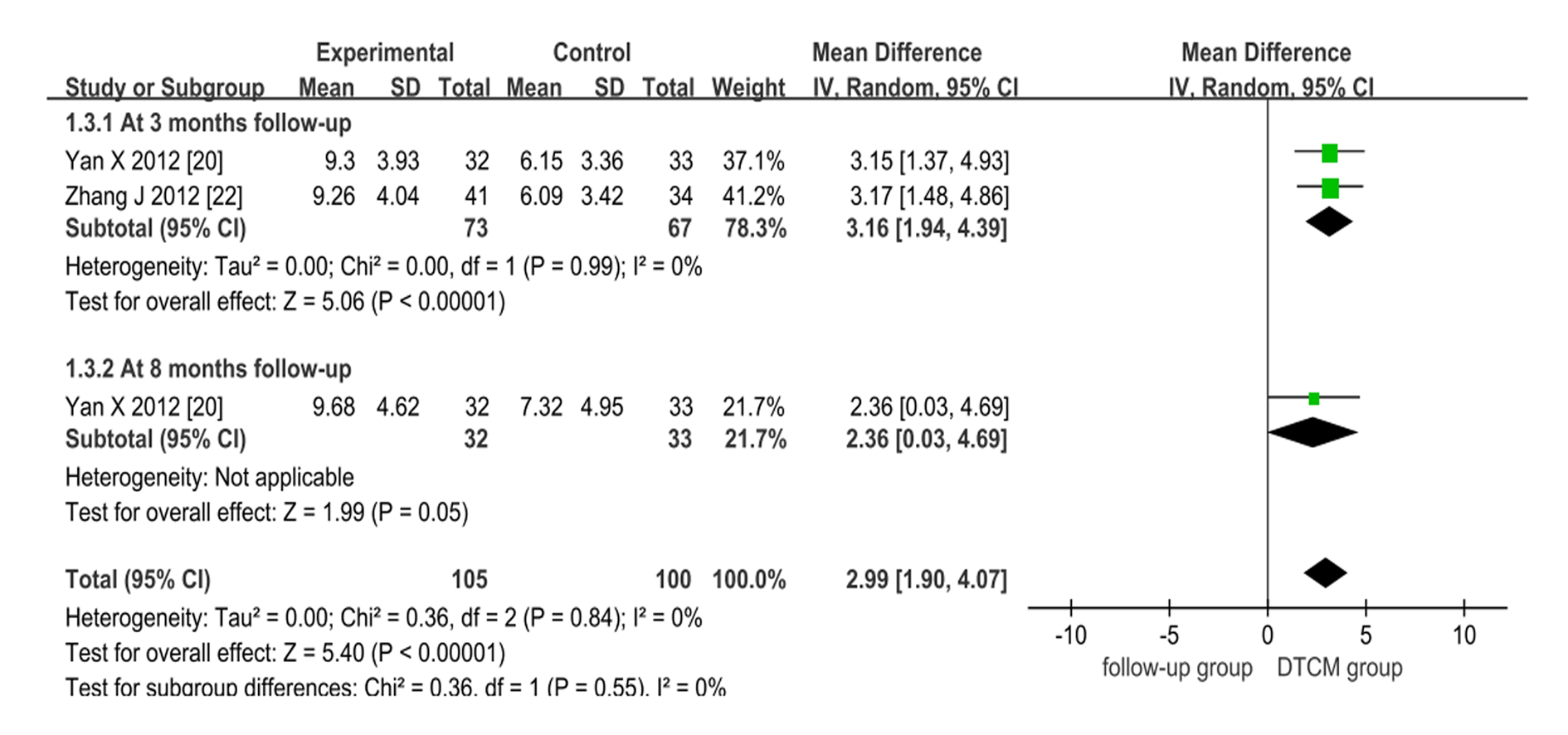

Supplement: S1 Fig — (TIF) [file pone.0213062.s006.tif]

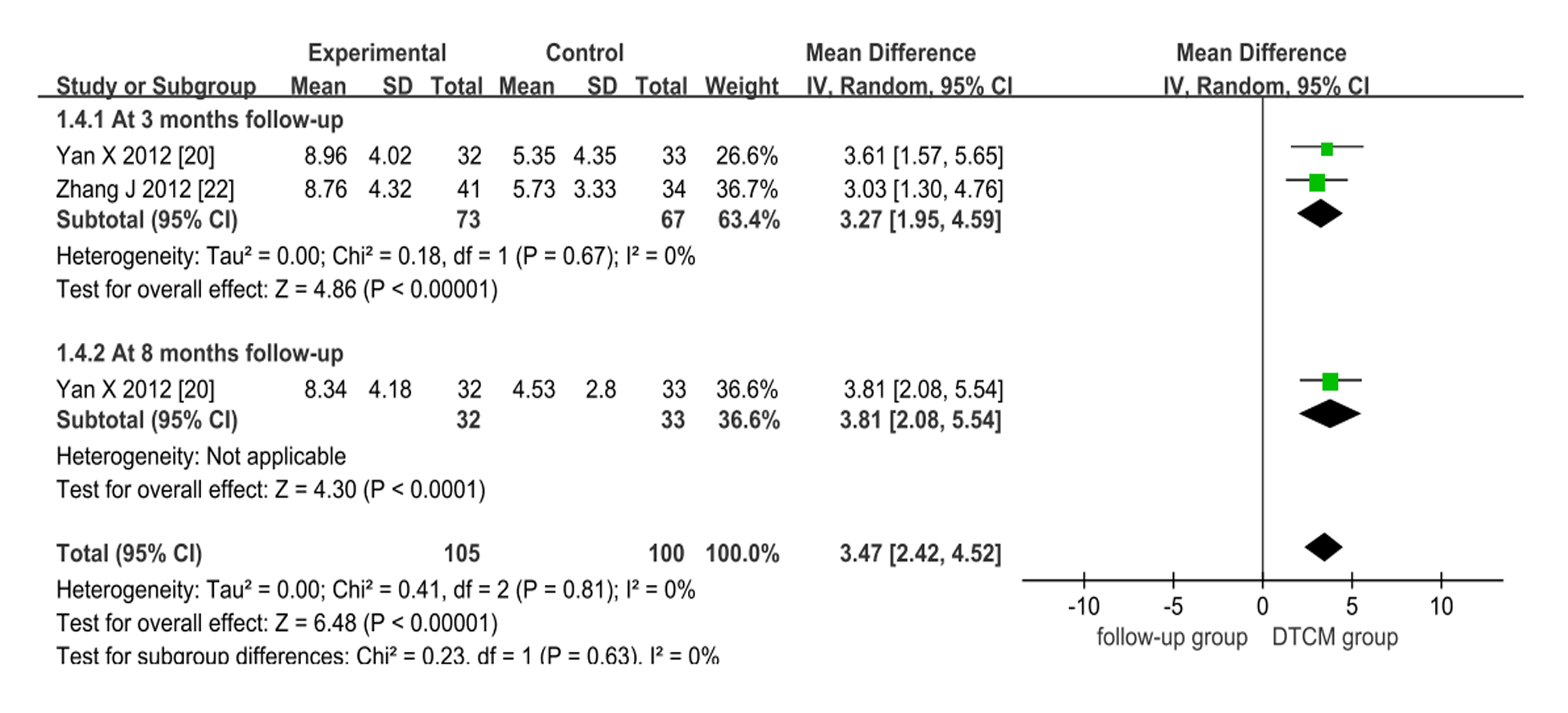

Supplement: S2 Fig — (TIF) [file pone.0213062.s007.tif]

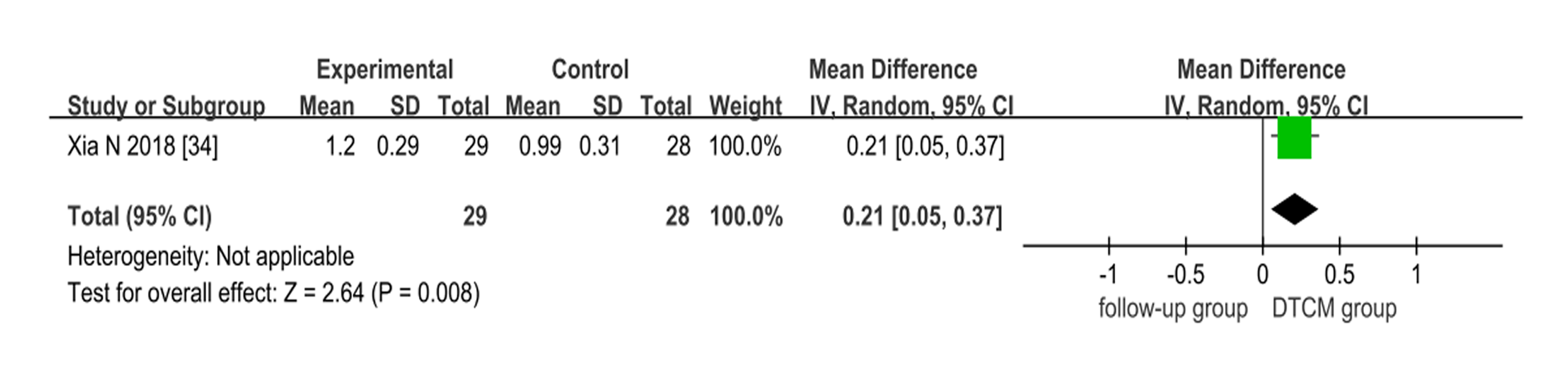

Supplement: S3 Fig — (TIF) [file pone.0213062.s008.tif]

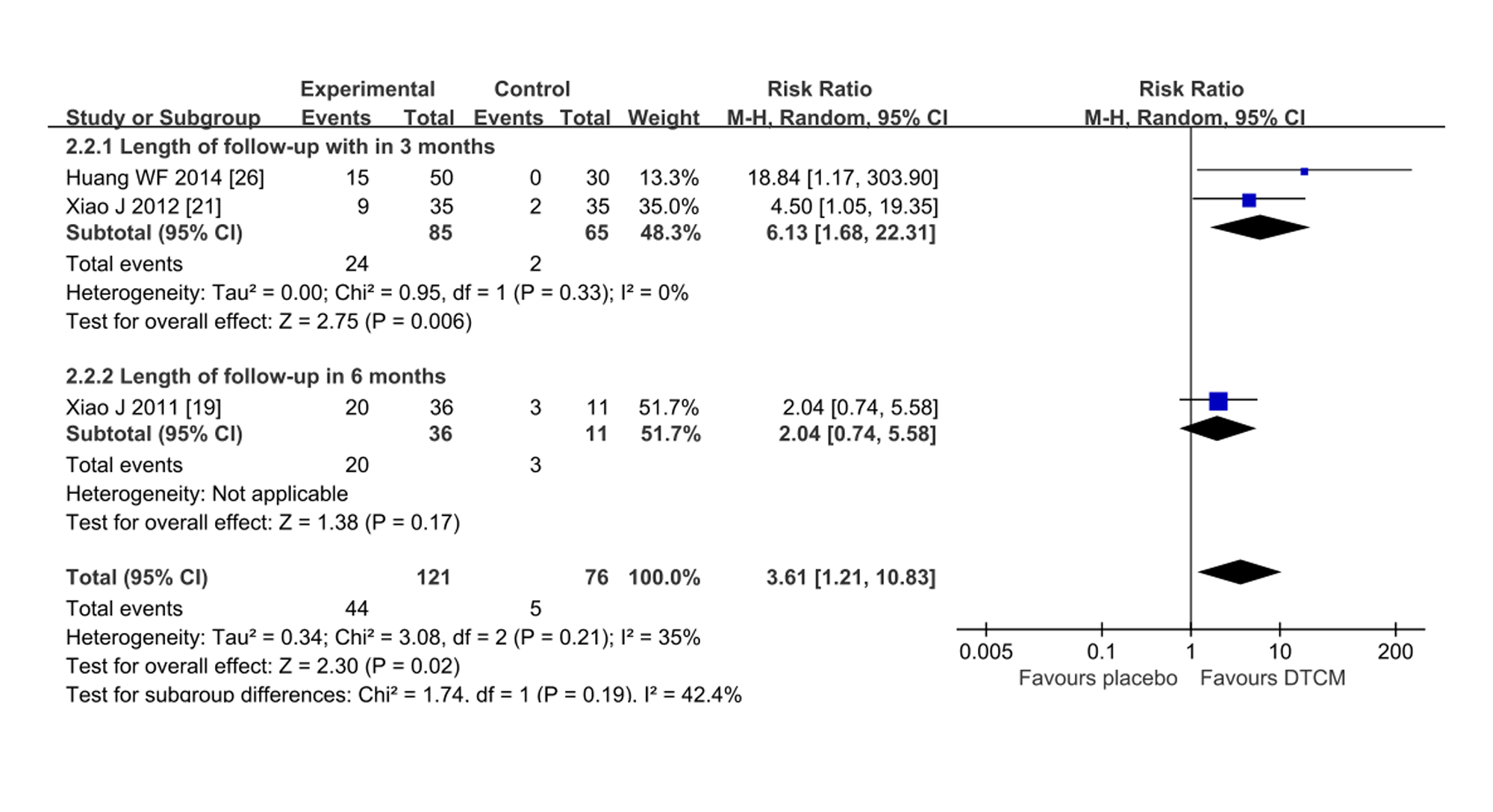

Supplement: S4 Fig — (TIF) [file pone.0213062.s009.tif]
